# On the Hunt for Chiral Single-Atom Catalysts

**DOI:** 10.1021/acscatal.4c07405

**Published:** 2025-04-12

**Authors:** Theodore A. Gazis, Vincenzo Ruta, Gianvito Vilé

**Affiliations:** † Department of Chemistry, Materials, and Chemical Engineering “Giulio Natta”, Politecnico di Milano, Piazza Leonardo da Vinci 32, IT-20133 Milano, Italy

**Keywords:** single-atom catalysis, asymmetric synthesis, heterogeneous catalysis, catalyst design, chiral catalysis

## Abstract

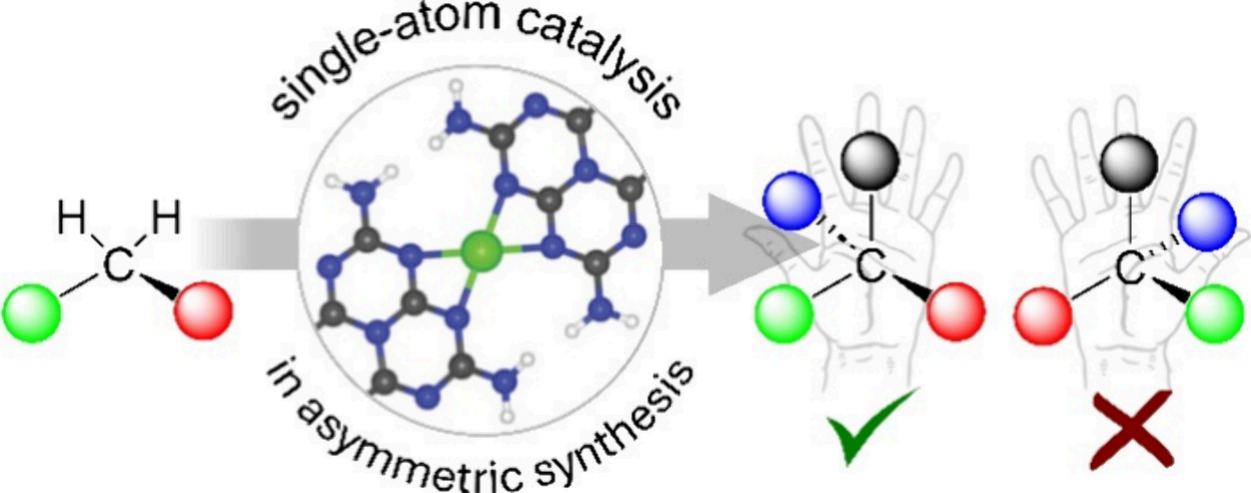

Enantioselective transformations are crucial in various fields, including chemistry, biology, and materials science. Today, the selective production of enantiopure compounds is achieved through asymmetric homogeneous catalysis. Single-atom catalysts (SACs) are emerging as a transformative approach in chemistry, enabling the heterogenization of organometallic complexes and effectively bridging the gap between homogeneous and heterogeneous catalysis. Despite their potential, the integration of SACs into enantioselective processes remains an underexplored area. This perspective offers a comprehensive analysis of possible strategies for the design of heterogeneous asymmetric catalysts, examining how chiral surfaces, chiral modifiers, grafted chiral complexes, and spatial confinement techniques can be effectively employed to enhance enantioselectivity. Each of these methods presents distinct advantages and challenges; for example, chiral surfaces and chiral modifiers offer potential for tailored reactivity but can suffer from limited stability and selectivity, while grafted chiral complexes provide robust platforms but may face issues related to scalability and synthesis complexity. Spatial confinement strategies show promise in enhancing catalyst efficiency but may be constrained by accessibility and reproducibility concerns. These strategies lay the groundwork for their adaptation to SACs, by providing innovative approaches to replicate the well-defined chiral environments of homogeneous catalysts while preserving the stability, reusability, and unique advantages of single-atom heterogeneous systems.

## 1 Introduction

Chirality plays a pivotal role in molecular interactions, fundamentally influencing how molecules are recognized and behave in chemical and biological processes. At its core, it refers to the geometric property of a molecule having a non-superimposable mirror image, akin to the difference between left and right hands. This seemingly subtle distinction can dramatically influence their activity and properties, making the study and application of chirality crucial in various scientific and industrial domains.^1^ In chemistry, the significance of chirality was highlighted by Louis Pasteur in the beginning of the 19th century when he discovered that certain compounds could exist as mirror-image forms, named enantiomers.^2^ Despite having identical chemical compositions, enantiomers can exhibit vastly different behaviors in biological systems. A stark example is thalidomide, a drug initially marketed as a sedative and used to treat morning sickness in pregnant women. Tragically, while one enantiomer had the desired therapeutic effect, its mirror image caused severe birth defects, demonstrating the importance of chirality in drug design and development.^3^ Crucially, chirality’s importance extends beyond organic molecules, influencing materials science, where it imparts distinctive optical, electronic, and mechanical properties. For instance, chiral silicon enhances efficiency through direct emission of polarized light, a crucial consideration for photonic devices. Additionally, chiral silicon is employed in biocompatible sensors to detect chiral biomolecules, advancing medical diagnostics and personalized medicine.^4^

To address the challenge of controlling and utilizing chirality, particularly in medicinal chemistry, significant advancements have been made in chemical synthesis. A primary objective is the selective generation of a specific enantiomer, a process known as asymmetric synthesis. Central to this process is the use of chiral catalysts, designed to induce the formation of a specific enantiomer with high precision.^5^ As catalysts can exist in both homogeneous and heterogeneous forms, the development of both homogeneous and heterogeneous asymmetric catalytic processes is an important research direction. In this context, homogeneous chiral catalysts offer high enantioselectivity and activity with well-defined active sites. However, they are costly to design, can be sensitive to air and moisture, and are difficult to separate, leading to poor recyclability.^1^ In contrast, heterogeneous chiral catalysts are easier to separate, reusable, and more stable.^1^ However, their development faces significant challenges intrinsic to solid catalysts, including lower selectivity and activity compared to homogeneous variants, and reproducibility issues. This can be attributed to the irregular arrangement of active sites, which fails to provide the uniformity necessary for high activity and enantioselectivity. Additionally, the preparation and characterization of heterogeneous catalysts is complex, requiring extensive experimentation and fine-tuning to optimize.^1^

To address these limitations, there has been a growing shift toward the development of single-atom catalysts (SACs). This emerging class of catalysts consists of individual metal atoms dispersed on a support material, as opposed to forming clusters or nanoparticles (NPs). Due to their atomic-scale design and efficient metal usage, SACs possess well-defined active sites akin to homogeneous catalysts. Moreover, SACs retain the key advantages of heterogeneous catalysts, such as easy separation from reaction mixtures and improved recyclability. Taking it a step further, the strong metal–support interactions in SACs enhance catalyst stability and prevent metal aggregation - an issue often encountered in conventional heterogeneous catalysts.^6^ Current research has established SACs as effective catalysts for small molecule activation such as H_2_, O_2_, and CO_2_ often surpassing their homogeneous counterparts.^7,8^ On the other hand, their application in more complex transformations under liquid phase conditions remains in its early stages. Nonetheless, SACs hold significant potential across various fields, from organic synthesis to pollutant degradation and waste valorization. These applications exploit not only traditional thermal approaches but harness enabling technologies, such as photo- and electrochemistry.^9,10^ Also hybrid approaches have been explored, by anchoring single metal atoms onto enzymes to enable efficient catalysis under ambient conditions. In this case, the ability of the enzyme’s active pocket to recognize and bind reaction intermediates lowers the activation energy of metal-catalyzed reactions, leading to enhanced catalytic performance.^11^

Despite the promise of SACs and the importance of chirality, only a limited number of reports successfully marry these two areas. This scarcity is mainly due to technical difficulties in the synthesis of SACs, where ensuring precise atomic dispersion and stabilization on the support is critical. Introducing enantioselective regions into these systems adds another layer of complexity to an already intricate synthetic process. Furthermore, characterizing SACs requires advanced techniques, such as high-angle annular dark field-scanning transmission electron microscopy (HAADF-STEM) and extended X-ray absorption fine structure (EXAFS) spectroscopy to verify atomic dispersion.^12^ Chiral SACs will demand even more specialized techniques rarely employed in conventional catalysis including, chiral surface plasmonic resonance (CSPR),^13^ circular dichroism,^14^ and scanning-tunnelling microscopy (STM).^15^

**Figure 1:**
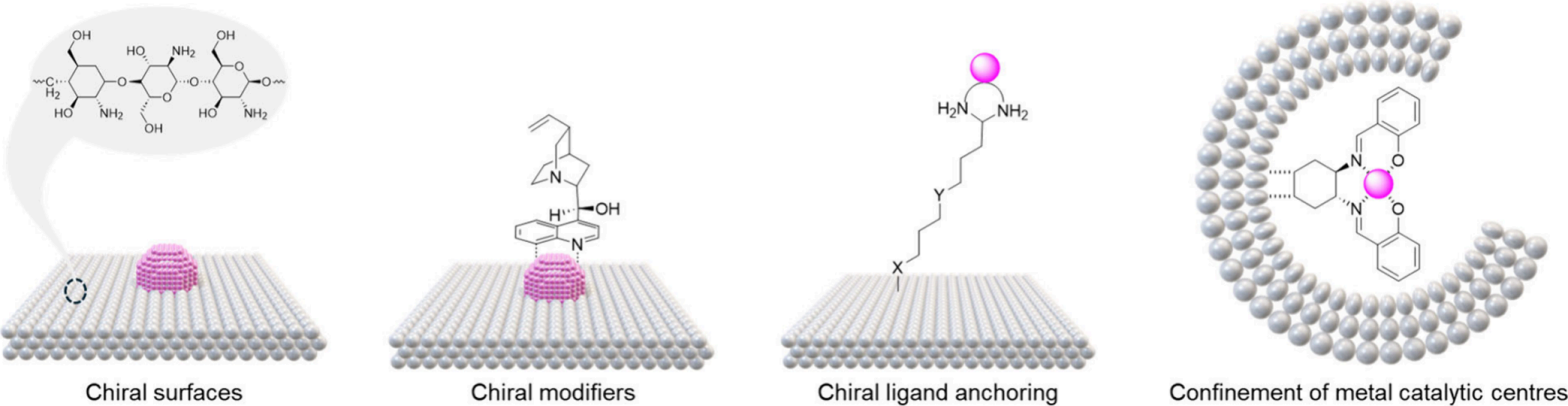
Key design strategies for introducing and controlling chirality in catalysts. These approaches are pivotal for precisely tailoring chiral environments at the atomic level, thereby also enhancing the selectivity and efficiency of SACs in asymmetric catalysis and enabling the production of optically pure compounds via single-atom catalysis.

Another important factor is the inherent complexity of the asymmetric heterogeneous catalysis field. While extensive research has been conducted, there is little cohesion between the reported studies. For the uninitiated, navigating this fragmented landscape can be daunting. This perspective thus aims to provide an introductory overview of the current design strategies for heterogeneous asymmetric catalysis and explore how these systems can be adapted to develop chiral SACs. For the scope of this perspective, we focus on four key design approaches, as illustrated in Figure 1: (*i*) inherently chiral surfaces, (*ii*) chiral modifiers, (*iii*) anchoring of chiral complexes, (*iv*) spatial confinement methods. Each approach is critically analyzed, in the context of its effectiveness, challenges, and potential for adaptation to SACs. For each design principle, we showcase the breadth of reactions solid-state asymmetric catalysis have been applied to, as well as the chiral characterization techniques available in the arsenal of the surface scientist. The concluding section provides a perspective on how the above-mentioned design principles can be applied to SACs, offering guidance to researchers interested in extending the reach of SACs to chiral catalysis.

## 2 Chiral Catalyst Design and Their Application in Asymmetric Reactions

### 2.1 Chiral Surfaces

The most intuitive and straightforward design for a solid enantioselective catalyst is simply using an innately chiral surface, without the need for additives to induce enantioselectivity in the substrate. While a variety of surfaces, including oxidic minerals and polymers, lack a center of symmetry and are thus chiral, they are rarely catalytically active. Additionally, although bulk metal surfaces are typically achiral, single metal crystals do exhibit chirality. However, large-scale preparation of chiral metal surfaces remains a significant challenge.^16^ Most importantly, enantioselectivity on such surfaces is not solely a function of their chirality but also dependent on enantiospecific interactions between the surface and the prochiral substrate.^17^ These interactions are influenced by factors such as molecular adsorption, surface coverage, and homogeneity, all of which contribute to consistent enantioselective binding. Given the small energy difference between enantiomers, computational modeling of surface-substrate interactions is challenging.^18^ These factors collectively make the identification of a surface capable of both inducing enantioselectivity and serving as a catalyst for a specific substrate a labor-intensive process, often reliant on experimental trial and error.

**Figure 2:**
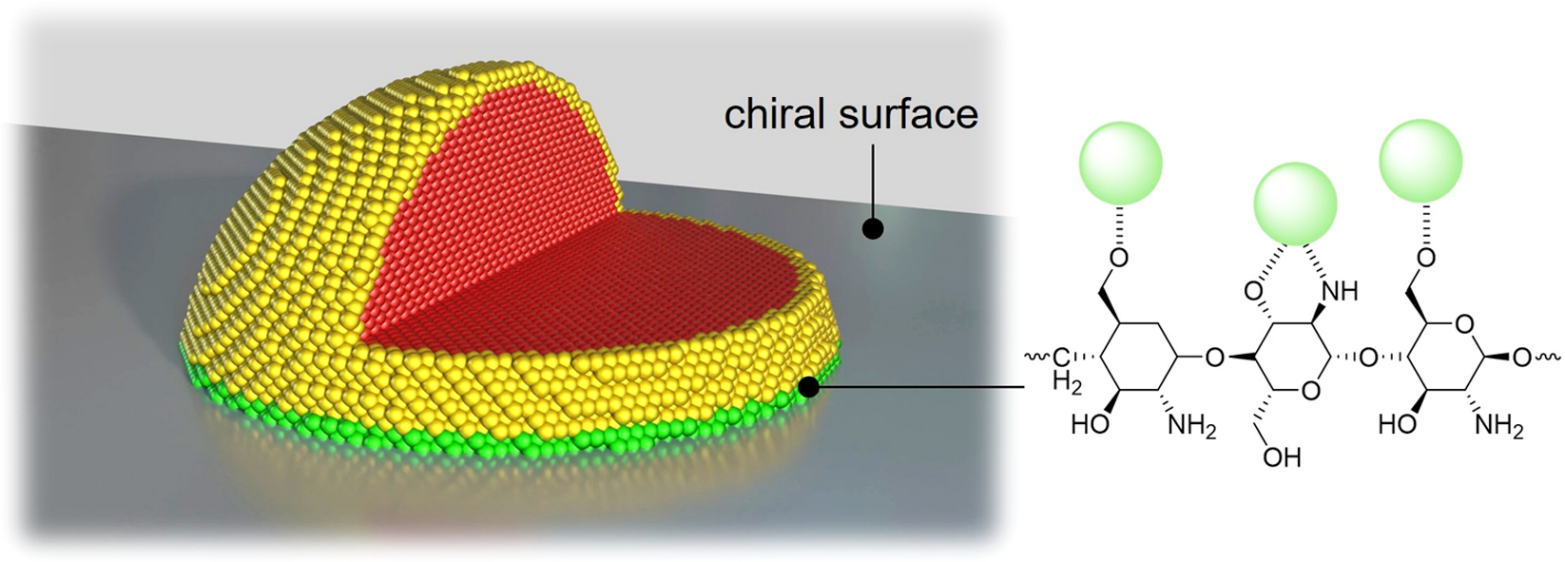
Schematic representation of a metal nanoparticle on an intrinsically chiral surface. Only the atoms in contact with the asymmetric support (green) among the catalytically active sites (green and yellow) can convey chiral information. Internal atoms (red) are inactive both catalytically and chirally.

A potential solution involves separating the roles of catalysis and enantioselectivity by immobilizing a catalytically active metal or metal oxide onto an innately chiral surface. This combination is expected to enhance catalytic activity and ensure that chiral information is effectively transferred to the substrate. A 1932 study by Schwab and Rudolph was among the first to explore the concept, utilizing naturally chiral quartz as a scaffold for Cu, Ni, and Pt catalysts.^19^ Their work demonstrated a preference for one enantiomer in the dehydrogenation of racemic 2-butanol. Although the enantiomeric excess (ee) achieved was below 10%, their research provided a critical proof of concept for this methodology. Consequently, this approach has been expanded to other hydrogenation/dehydrogenation protocols and adapted to alternative chiral supports such as silk, fibroin, or cellulose. Notable examples include Pt and Pd catalysts on chitosan and wool reportedly affording up to 100% ee in the hydrogenation of simple ketones and a Pd@silk catalyst purportedly furnishing 66% optical yield in the hydrogenation of benzylidene oxazolidone.^20,21^ Crucially, none of these reports proved reproducible, a factor attributed to the harsh reaction conditions required for metal deposition onto the support. Follow-up reports indicated the strongly acidic conditions used during Pd deposition onto silk could degrade the support. It was postulated that this decomposition had a dual effect: the degradation products acted as chiral modifiers (Section 2.2) and contributed to the reproducibility issues. Despite initial promise, broader implementation of natural chiral supports in enantioselective catalysis remains limited. This scarcity arises as only metal atoms in direct contact with the support can impart chirality to the substrate. However, the majority of exposed metal sites are too far from the support, leading predominantly to racemic products, as represented in Figure 2. Theoretical studies have corroborated this limitation, proposing that the growth of thin metal films could present a viable solution by enhancing the proportion of chiral-active metal sites.^22^

The inherent limitations of innately chiral supports mean that recent focus has shifted to synthetic chiral surfaces. Among these, chiral metal–organic frameworks (MOFs) and covalent organic frameworks (COFs) are particularly prominent due to their predictable design, high porosity, and structural flexibility.^23,24^ A key concept in the design of chiral MOFs and COFs is chiral amplification, where a small initial chirality, typically introduced during synthesis via chiral ligands or building blocks, propagates throughout the entire framework, making the structure globally chiral. This amplified chirality enhances the material’s ability to selectively interact with one enantiomer over another, improving enantioselectivity in catalysis. Additionally, spatial confinement of catalytically active species within the pores of these frameworks further enhances enantioselectivity by restricting reactant movement, forcing them to adopt specific orientations which stabilize transition states and improve enantioselective control. In short, chiral amplification and spatial confinement work synergistically to achieve high enantioselectivity, and these concepts will be discussed in greater detail in Section 2.4.

### 2.2 Chiral Modifiers

Chiral modifiers are some of the most extensively studied and widely utilized components in asymmetric heterogeneous catalysis, particularly in the context of hydrogenation reactions.^25^ This approach owes its popularity to its simplicity, as it allows the use of standard achiral surfaces decorated with metal nanoparticles. Instead, the solid catalyst is pretreated with soluble optically active molecules which adsorb onto the metal catalyst surface, creating a localized chiral environment. This approach has been shown to induce enantioselectivity comparable to homogeneous processes with the added benefits of easier catalyst recovery and reusability.

Key factors for designing efficient catalytic systems include the molecular structure of the chiral modifier and prochiral substrate, the properties of the heterogeneous catalyst, the choice of solvent and reaction conditions used. A list of commonly used chiral modifiers for heterogeneous asymmetric catalysis is displayed in Table 1, alongside their cost, commercial availability, and boiling point.

**Table 1:** Commonly Encountered Chiral Modifiers for Asymmetric Catalysis and Their Key Properties and Cost

Name	Commercial availability	Boiling point (°C)	Cost (€ g^–1^)^a^	ref
Cinchonidine (CD)	Yes	427^26^	6.79	(27)
10,11-dihydrocinchonidine	No	n.a.^b^	n.a.	(28)
*O*-3,5-bis(trifluoromethyl)phenyl-cinchonidine	No	n.a.	n.a.	(29)
*O*-(3,5-dimethylphenyl)-cinchonidine	No	n.a.	n.a.	(29)
*O*-phenylcinchonidine	No	n.a.	n.a.	(29)
*O*-methyl cinchonidine	No	n.a.	n.a.	(66)
Cinchonine	Yes	427^30^	1.99	(27)
(+)-10,11-dihydrocinchonine	Yes	n.a.	12280	(28)
(−)-quinine	Yes	463^31^	2.96	(32)
l-proline	Yes	252^33^	2.23	(37)
Diethyl L-tartrate	Yes	280^34^	0.64	(28)
L-(+)-tartaric acid	Yes	169–172^35^	0.53	(35)
(*S*)-(−)-1,1′-bi(2-naphthol) (*S*-BINOL)	Yes	387^36^	1.60	(37)
(1*S*,2*S*)-(+)-1,2-diaminocyclohexane	Yes	104–110^38^	86.40	(37)
(*R*)-(+)-1-(1-naphthyl)ethylamine	Yes	153 (at 11 mmHg)^39^	13.40	(40)
Benzylamine	Yes	185^41^	0.09	(42)
(*R,S*)-pantoylnaphthylethylamine	No	n.a.	n.a.	(43)
(5*S*)-2,2,3-trimethyl-5-phenylmethyl-4-imidazolidinone (MacMillan catalyst; various salts)	Yes	n.a.	102.50	(44)

a Obtained from Merck.

b Not available.

Two major families of chiral modifiers dominate this chemical space, distinguished by their distinct mechanisms of chirality transfer to the substrate. The first employs tartaric acid, typically in conjunction with nickel nanoparticle catalysts. Due to its simplistic structure, a single molecule of tartaric acid alone cannot create a sufficiently detailed localized chiral environment. Instead, multiple tartaric acid molecules are thought to interact with the substrate through hydrogen bonding, selectively favoring one enantiotropic face. Experiments with model surfaces, such as 2-butanol and propylene oxide on palladium and platinum, support this notion.^45^ The scope of this mode of chiral inducement is limited to a narrow set of reactions, a key example of which is the enantioselective hydrogenation of β-ketoesters and β-diketones. Despite its niche application, tartaric acid continues to draw academic interest due to its very low cost and ease of use.

**Figure 3:**
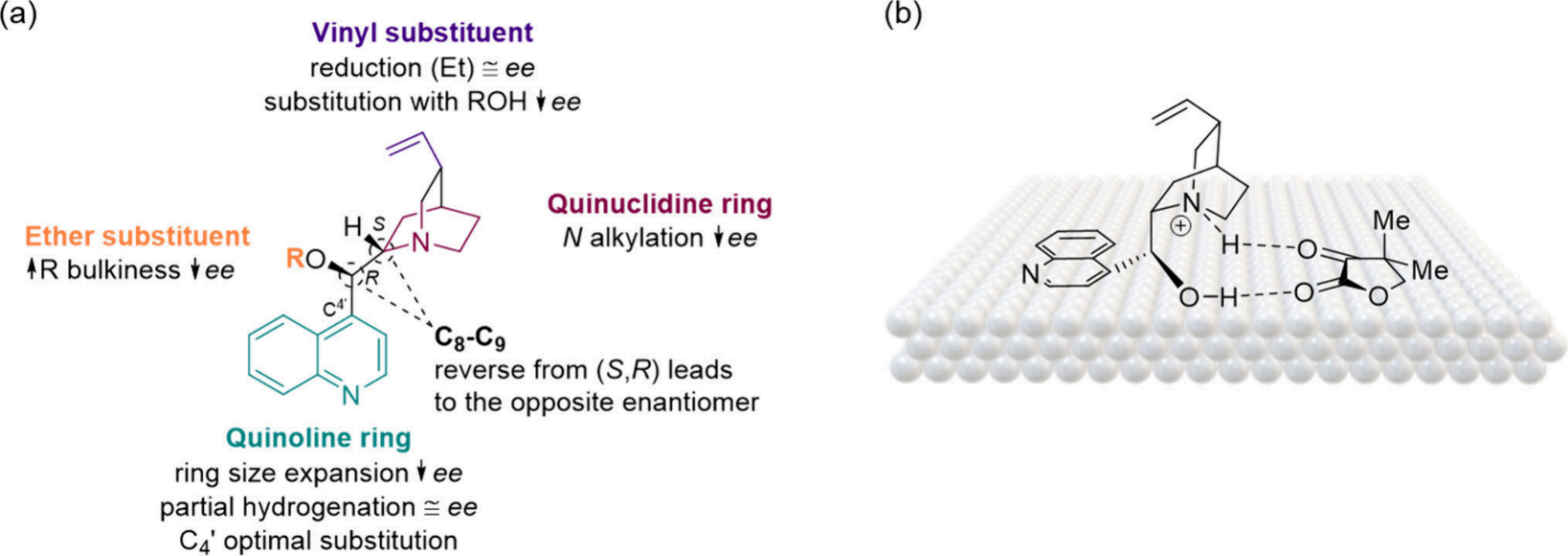
Cinchonidine-based chiral modifier structure–activity relationship (a); binding mode of cinchonidine on a generic supported metal nanoparticle (b). Adapted from ref (46, 47).

In the second system, noble metal (Pt, Rh, Ir, and Pd)-based catalysts are used in conjunction with cinchona alkaloids as the chiral modifiers. The latter possess three key structural features: an aromatic moiety for adsorptive anchoring to the support surface, a stereogenic inducing region, and an interactive moiety to direct the prochiral substrate (Figure 3).^45−47^ Thanks to these features, these modifiers have been extensively applied in asymmetric hydrogenations with enantioselectivities rivalling those of homogeneous catalysts.^48^

Unlike tartaric acid, cinchonidine and its derivatives do not rely on the formation of supramolecular aggregates on the catalyst surface to induce chirality. Instead, each individual molecule independently creates a localized chiral environment. This intrinsic ability, combined with the highly modular framework of cinchonidine, enables precise structural modifications to develop detailed structure–activity relationships and optimize catalytic performance. As a result, cinchona alkaloids have become versatile tools for targeting a diverse range of substrates and remain the most widely employed chiral modifier in asymmetric hydrogenations and beyond.

The conformation of cinchona alkaloids, shaped by factors such as temperature,^49^ solvent,^50^ and substituents,^27^ directly affects their interaction with the catalyst surface and substrates, thereby influencing the stereochemical outcome of reactions. Structural studies highlight the importance of substituents on the C_8_–C_9_ carbon, where bulky groups often decrease enantiomeric excess and can even cause enantioselectivity reversal.^29^ In a similar vein, rigid *O*-ethers at these positions exhibit distinct kinetic behavior and tend to lower substrate enantioselectivity compared to their more flexible counterparts.^32^ On the other hand, fluorine substituents at C_9_ maintain high performance.^51^ Drastically altering the substituents to imidazolidinone, proline-based derivatives^47^ or even peptides derived from tryptophan^52^ show potential but varied effectiveness compared to the parent cinchona alkaloids. Mandelic acid derivatives can also act as modifiers, albeit with moderate enantioselectivity due to weak substrate adsorption.^53^

In asymmetric hydrogenations, cinchona alkaloids exhibit a clear preference for adsorption on noble metals, with a clear division between carbonyl and alkene hydrogenations. Carbonyl hydrogenations are predominantly carried out using Pt nanoparticles, which demonstrate size- and shape-dependent catalytic performance. Pt NPs approximately 3 nm in size are alleged to exhibit optimal activity and enantioselectivity.^54^ Studies by Schmidt et al. revealed the shape sensitivity of Pt nanoparticles in the hydrogenation of ethyl pyruvate (EP) and ketopantolactone (KPL), where different nanoparticle morphologies - such as cubic, cuboctahedral, and octahedral - demonstrated distinct catalytic behaviors.^55^ The same study further concluded that ideal catalysts for activated ketones should predominantly feature Pt(111) terraces.

Additionally, the use of other noble metals like Rh, Ir and Ru has been explored and shown to display similar enantioselectivity dependence to Pt. For instance, Hoxha et al. investigated Rh@Al_2_O_3_ catalysts with narrow size distributions for the enantioselective hydrogenation of EP and ethyl 3-methyl-2-oxobutyrate, using quinine as a chiral modifier.^56^ They found that both the enantioselectivity and turnover frequency decreased with smaller Rh particle sizes (0.9 to 1.7 nm). Similarly, studies have shown that larger Ir particles improved both conversion and enantioselectivity in the hydrogenation of EP, *p*-phenylenediamine (PPD), and acetophenone.^57^ The oxidation state of the metal is another crucial factor, with Ir^δ+^ species enhancing reaction rates, a phenomenon linked to the polarization of the carbonyl bond.^58^ Finally, although less extensively studied, Ru catalysts have demonstrated potential in enantioselective hydrogenation. Ye et al. prepared various oxide-supported Ru catalysts, modified with PPh_3_ and chiral diamines, for aromatic ketone hydrogenation.^59^ Their study established a clear correlation between increasing support basicity (MgO > Al_2_O_3_ > CeO_2_ > ZnO > SiO_2_) and enantioselectivity across all prochiral substrates: in this case, the nucleophilic groups on the oxide surface—the oxygen anions (O^2–^) and surface hydroxyl groups (−OH) commonly present in metal oxides—interacted with the electrophilic carbon of the carbonyl group. Therefore, the properties of the support play a critical role, by both influencing the electronic properties of the NPs as catalytic centers, and enhancing the adsorption of the chiral modifier on the catalytic surface. While increasing support basicity appears advantageous for Ru NPs, Pt NPs typically perform better on acidic supports. In this regard, aluminum oxide has proven particularly effective. Hoxha et al. demonstrated that adjusting the acidity of the alumina support through silica addition improved enantioselectivity in the hydrogenation of keto-phenylalanine. This was attributed to the modification of Pt electronic properties, which are influenced by the acidity of the alumina support, ultimately affecting the Pt–H interaction as suggested by CO adsorption studies.^60^ In contrast, basic supports like cesium oxide negatively impacted performance. Composite supports have also proven effective, with Li et al. showcasing the utility of Pt catalysts supported on alumina-carbon composites in the highly enantioselective hydrogenation of ethyl 2-oxo-4-phenylbutyrate.^61^ In this case, introducing alumina into the carbonaceous support resulted in three effects: firstly, it stabilized the mesostructure of carbon, leading to the formation of relatively large pores that enhanced the diffusion and adsorption of the chiral modifier onto the catalyst surface. Secondly, alumina incorporation into the mesopores of the carbon host facilitated the generation of Pt^δ+^ species, which are essential for enantioselective catalysis. Thirdly, in acetic acid solvent, alumina can form an electrophilic species, (Al(OAc)_2_)_3_O^+^, which promotes the adsorption of the chiral modifier.

Several studies explore additives and comodifiers in asymmetric hydrogenation. Szöri et al. found trifluoroacetic acid significantly enhanced ee from 50% to 92% in 2,2,2-trifluoroacetophenone (TFAP) hydrogenation with CD-modified Pt.^62^ Molecular studies suggested trifluoroacetic acid (TFA) interacts with CD and substrate, forming crucial hydrogen bonds. Sano et al. observed up to 93% ee improvement with 1% ionic liquids (ILs) in methyl benzoylformate (MBF) hydrogenation, affecting Pt interaction and reaction rate.^63^ Tálas et al. showed tertiary and secondary amines enhance ee and reaction rate in EP and MBF hydrogenation, by altering the rate of CD adsorption.^64^

Chiral modifier-induced asymmetric hydrogenations of C = C bonds, especially olefins, have been widely explored, albeit to a lesser extent compared to carbonyl hydrogenations.^65,66^ In this context, chirally modified Pd catalysts have been a focal point, showing high enantioselectivity for substrates like phenyl cinnamic acid (PCA) and various aliphatic acids.^67^ Research has emphasized the impact of Pd particle size and shape on catalytic performance: larger Pd nanoparticles favor better enantioselectivity due to their flat surface planes, while smaller particles with more edge sites promote racemic product formation.^68,69^ The structure of the support material also plays a crucial role. For instance, the high surface area and two-dimensional structure of graphene promote the uniform dispersion of metal nanoparticles, enhancing their interaction with chiral modifiers. Additionally, its excellent electrical conductivity facilitates electronic interactions with the metal centers, effectively modulating their electronic properties. In contrast, TiO_2_ exhibits strong metal–support interactions, which can alter the oxidation state and electronic characteristics of the supported nanoparticles. Its surface, enriched with oxygen vacancies and hydroxyl groups, provides specific adsorption sites for chiral ligands, thereby contributing to the formation of a well-defined chiral environment essential for enantioselective catalysis.^70,71^ Amine additives, like benzylamine, improve enantioselectivity by enhancing the desorption of the product, thus promoting efficient catalysis.^72,73^ Once more cinchona alkaloids show exceptional enantioselectivity for certain substrates, and its interaction with the prochiral substrate on the Pd surface determines the degree of chiral induction.^72,74^ Additionally, (*S*)-proline has been explored as an alternative chiral modifier, particularly for asymmetric hydrogenation on Pd, influencing the enantioselective outcome through kinetic resolution mechanisms.^75,76^

**Figure 4:**
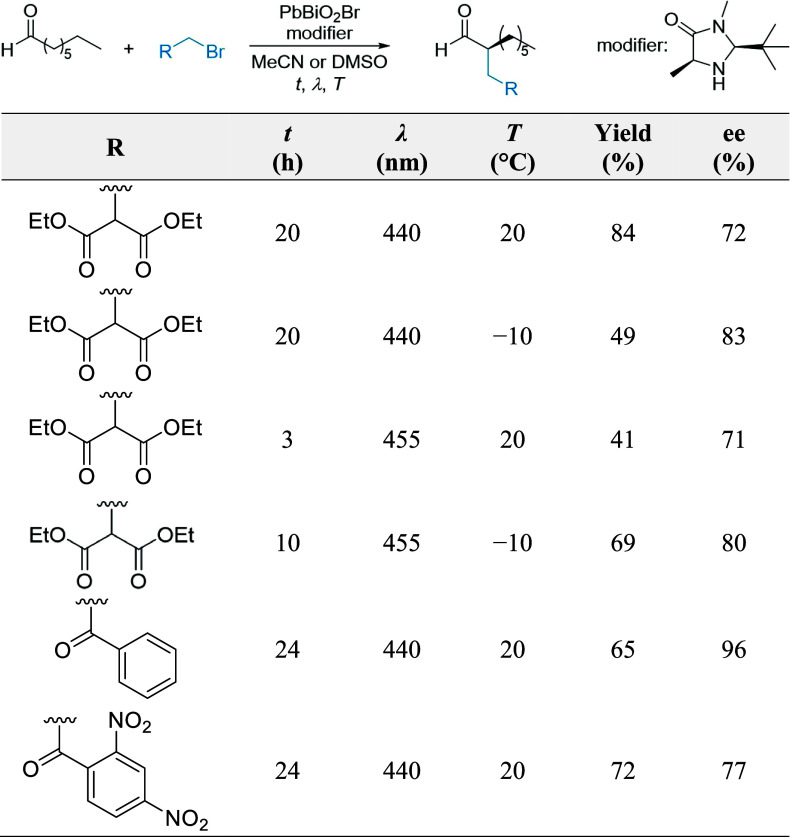
Photocatalytic α-alkylation of aldehydes using MacMillan’s organocatalyst as a chiral modifier, and PbBiO_2_Br as the catalyst. For each substrate, the optimal light wavelength (λ), temperature (T), and reaction time (t) have been reported. Adapted from ref (44).

While cinchonidine, its derivatives and to a lesser extent proline in combination with metal nanoparticles, are ubiquitous in asymmetric hydrogenations, other transformations offer greater flexibility. For instance, Cherevatskaya et al. utilized a MacMillan organocatalyst to screen PbBiO_2_Br and TiO_2_ based photocatalysts for the enantioselective α-alkylation of aldehydes (Figure 4). Under optimized conditions, yields of 90% and complete stereoselectivity for the desired product were achieved. Thus, this work elegantly demonstrates the synergistic potential of homogeneous organocatalysis with heterogeneous photocatalysis in asymmetric transformations.^44^

**Figure 5:**
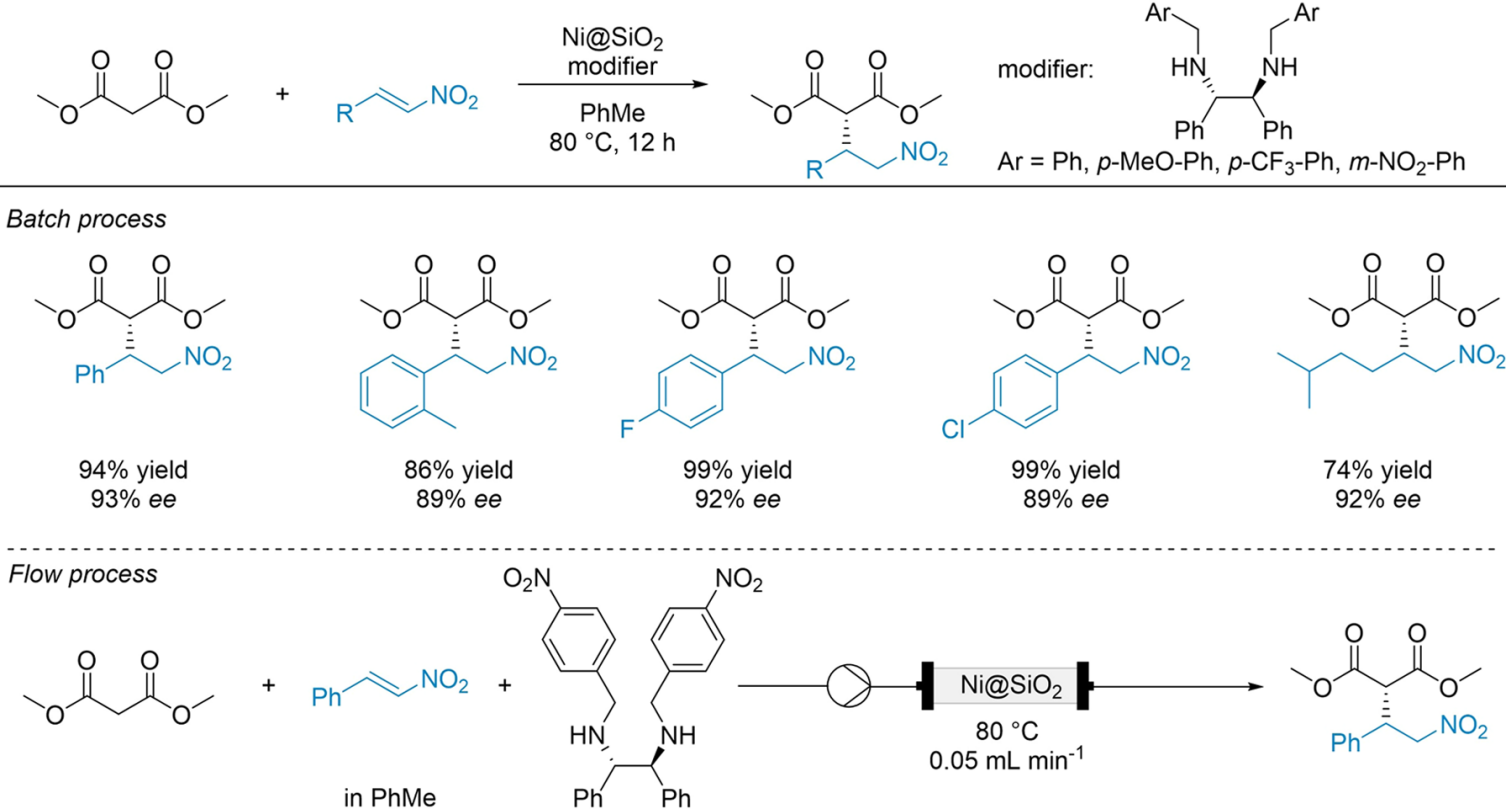
Diamine-modified Ni@SiO_2_ for the batch and continuous-flow alkylation of diethylmalonate with nitroalkenes. Adapted from ref (43).

All studies discussed thus far have been conducted in batch processes. As in achiral catalysis, transitioning to continuous-flow systems holds the promise of improved scalability (due to improved mass and heat transfer) better mixing conditions, precise control of reaction parameters, and the ability to safely handle hazardous chemicals.^77−79^ However, in the case of chiral catalysis, significant losses in catalytic activity and enantioselectivity have been observed. This is because in batch reactions the catalyst remains in contact with the reaction mixture for a longer period, enabling prolonged interaction between the catalyst, modifier, and substrate. Ren et al. successfully applied a chiral diamine-modified Ni@SiO_2_ catalyst, in the asymmetric Michael addition of dimethylmalonate to substituted nitroalkenes, as depicted in Figure 5.^43^ Under batch conditions, the catalyst outperformed its homogeneous counterpart, achieving over 99% yield and up to 93% ee, with structuring studies highlighting the role of Ni nanoparticle size and the importance of peripheral metal sites. However, adapting this system to continuous-flow conditions posed significant difficulties. Under continuous-flow conditions, it is likely that the chiral modified may bind less effectively or become more easily displaces from the catalyst surface due to the constant flux of reactants and products. This weaker, dynamic interaction reduces modifier coverage on the nanoparticles, ultimately leading to significant losses in catalyst enantioselectivity.

### 2.3 Chiral Ligand Anchoring

The adsorption of a chiral modifier onto a metal decorated support is a dynamic and reversible process. To maintain enantioselectivity, large excesses of the modifier are required. Consequently, the need to separate the product from the modifier renders the process often expensive and environmentally unsustainable. To advance to a fully heterogeneous process, stronger irreversible interactions between the chiral moiety and solid support are necessary. Interestingly, mounting evidence suggests some of the adsorbed chiral modifiers mentioned above align with this requirement. For example, the formation of tartrate anions was observed when modifying a nickel catalyst with tartaric acid to facilitate β-ketoester hydrogenations. These species, formed due to deprotonation during adsorption, are likely irreversibly bound onto the catalyst surface.^80−83^ Similarly, while cinchona alkaloid adsorption in solution is reversible, irreversible adsorption has been proven in the gas phase.^84,85^

Despite providing valuable insights, the applicability of these examples is constrained to a narrow set of cases. A more versatile alternative would be to modify chiral additives with linker groups, allowing them to irreversibly anchor onto the support. Immobilization is most achieved through covalent interactions, as the enhanced stability of these bonds positively impacts catalyst durability and recyclability. Furthermore, both inorganic and organic support can be readily functionalized to facilitate covalent attachment, making this approach broadly applicable. On the other hand, noncovalent bonding (electrostatic, van der Waals, ion exchange, etc.) often necessitates extensive modification, such as the incorporation of strongly polar (e.g., SO_2_^–^) or hydrogen bond-promoting groups, along with careful selection and positioning of any necessary counterions.^28^ Additionally, ensuring the irreversibility of noncovalent interaction requires precise control of reaction conditions to maintain catalyst stability and performance.

**Figure 6:**
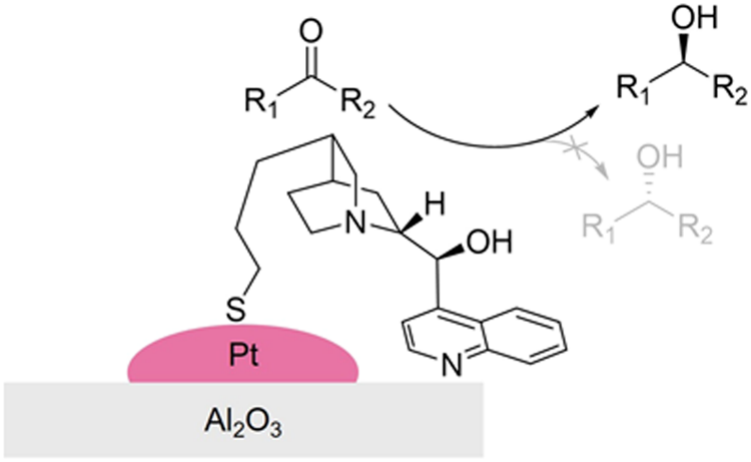
Cinchonidine-decorated Pt nanoparticles supported on Al_2_O_3_. Adapted from ref (86).

Though noncovalent approaches involve some inherent complexities, they remain a suitable option for solid enantioselective processes. An illustrative example involves coating the entire catalyst surface with thin layers of molecules that spontaneously organize themselves into a well-ordered structure. In the context of chiral catalysis, these self-assembled monolayers (SAMs) can be designed to incorporate chiral modifiers or ligands that impart a chiral environment on the surface of a catalyst. An illustrative example of this application involved decorating cinchonidine with alkanethiol anchoring groups of varying lengths.^86^ The chiral moiety of the catalyst was prepared by reacting the appropriate alkanedithiol with cinchonidine in the presence of azoisobutyronitrile (AIBN) as a radical initiator (Figure 6). Its chiral properties were established by NMR, FT-IR, and liquid injection field desorption ionization-mass spectrometry (LIFDI-MS). A simple liquid impregnation under inert atmosphere was sufficient to anchor it on Pt NPs supported on SiO_2_ and Al_2_O_3_ supports. The chiral adducts formed SAMs on the surface of the heterogeneous catalyst and could enantioselectively hydrogenate EP. In this instance, linker chain length represented a crucial factor, with longer chains demonstrating improved enantioselectivity but reduced activity due to increased ordering and limited surface accessibility. Conversely, shorter chains exhibited increased activity but were less effective at enhancing enantioselectivity. Medium-chain thiols offered the best balance, enhancing both metrics though failing to match the performance of a standard Pt@Al_2_O_3_ catalyst with cinchonidine in the liquid phase. Despite this shortcoming, SAM-modified catalysts represent a viable, fully heterogeneous alternative with satisfactory performance and operational advantages, particularly in terms of ease of catalyst separation. Beyond thiols, other functional groups have been applied as anchoring points for chiral SAMs, including phosphonate^87^ and silane^88^ moieties.

**Figure 7:**
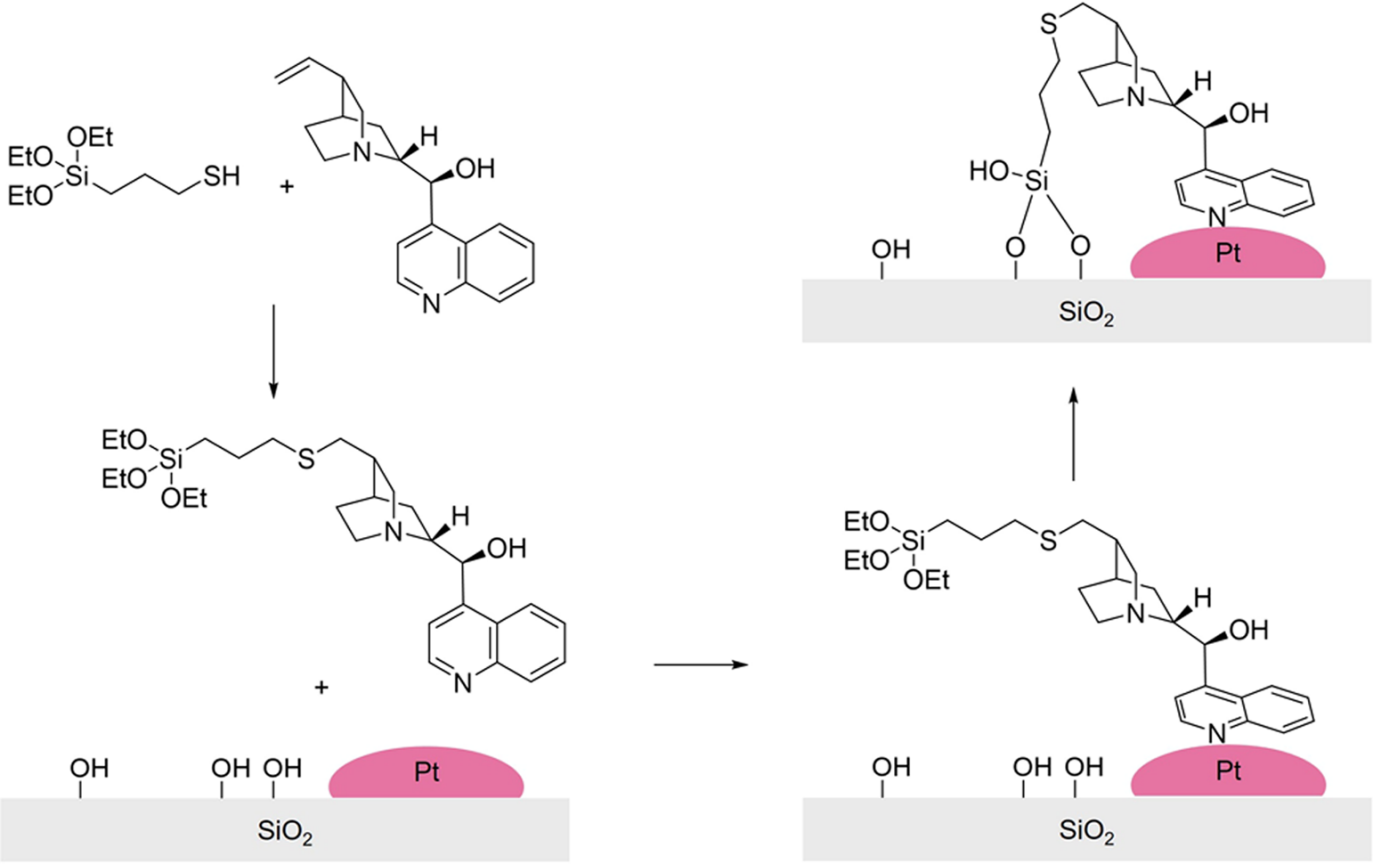
Schematic representation of the spatially controlled tethering of cinchona alkaloids next to Pt NPs dispersed on a high-surface-area silica support. Adapted from ref (90).

Covalent interactions offer a more targeted approach by grafting chiral moieties near the reactive sites of supported metal catalysts. This method utilizes the functional groups of surfaces as anchoring points, with oxide surfaces being particularly effective due to their terminal hydroxyl groups. Indeed, various well-established immobilization strategies including organosilane, phosphonate, carboxylate, and amine linkers can be utilized.^87^ Key considerations include the length and flexibility of the linker, the distance between the chiral site and the catalyst metal center as well as the nature of the attachment point. Each of these factors can profoundly influence catalyst activity and enantioselectivity, with this delicate interplay best illustrated in the work of Hong et al.^89^ In this study, cinchonidine was functionalized at two bonding points using AIBN as a radical initiator: a carbamate link to the alcohol position using 3-isocyanatopropyltriethoxysilane (ICPTEOS) and a mercapto bond at the vinyl position with 3-mercaptopropyltriethoxysilane (MerPTEOS). These functionalized species were grafted onto the silanol groups of Pt@SiO_2_ followed by a solvent flush to remove any excess or weakly adsorbed cinchonidine (Figure 7). Tethering through a carbamate bond improved performance compared to the mercapto bond. However, the enantioselectivity remained lower than free cinchonidine in solution, likely due to restricted mobility and poor surface coverage, with only 10% of surface hydroxyl groups derivatized. As a result, many Pt NPs lacked a nearby chiral environment. Additionally, the acidic hydroxyl sites on silica were catalytically active, causing racemic substrate conversion. Blocking surface hydroxyl groups with hexamethyldisilazane (HMDS) and using toluene to enhance silane polymerization can lead to improved (but still suboptimal) enantioselectivity. In response, the authors adopted a two-step catalyst synthesis approach. First, they leveraged the strong interaction between Pt nanoparticles and cinchonidine to form an adduct. Then, thermal activation was used to covalently tether the MerPTEOS linker to the silica, ensuring proximity between the stereodivergent and catalytic components. This resulted in superior activity and enantioselectivity, comparable to that of free cinchonidine in solution.

In the previous study, pretreating the metal center with a chiral modifier before immobilization demonstrated promise; however, this strategy requires careful optimization. This was illustrated by Watson et al., where they explored sulfur-containing chiral modifiers for the asymmetric hydrogenation of isophorone using Pd catalysts.^91^ Six chiral sulfide ligands with varying chain lengths were synthesized to anchor firmly onto Pd nanoparticles. The study revealed that larger alkyl groups on the sulfide improved enantiomeric excess by enhancing ligand dispersion. Despite this, catalytic activity and enantiomeric excess remained low, with the best ligand achieving only 14% enantiomeric excess. High-resolution XPS confirmed that ligand adsorption occurred exclusively on Pd nanoparticles, not on the carbon support. It was thus postulated that the presence of these thioether functionalities was responsible for poisoning the Pd catalyst, reducing the reaction rate by approximately 3 orders of magnitude compared to unmodified hydrogenation.

**Figure 8:**
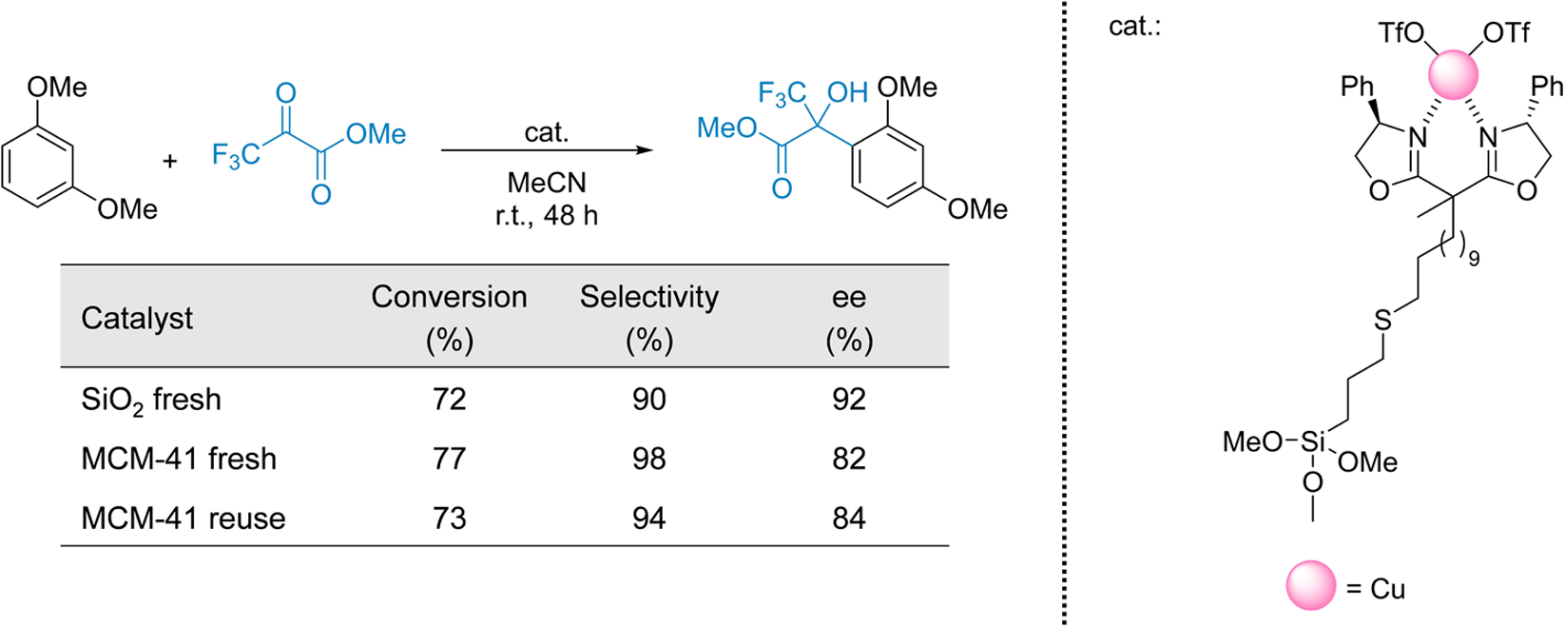
Immobilized Cu-bisoxazoline complex on SiO_2_ for the asymmetric Friedel–Crafts hydroxyalkylation of 1,3-dimethoxybenzene. Adapted from ref (92).

In the previous examples, heterogeneous catalysts were covalently modified by anchoring standard chiral modifiers, such as cinchonidine and its derivatives. An alternative approach involves the immobilization of chiral organometallic complexes, including metal-salen and metal-bisoxazoline complexes. This approach differs not only in structural aspects but also in the reaction mechanism. While the mechanisms for grafted modifiers resemble those described in Section 2.2, the reaction mechanisms in the presence of grafted organometallic complexes more closely align with those of their traditional homogeneous, non-supported counterparts. Nonetheless, the heterogenization of these complexes offers the potential advantage of reusability, as with grafted organometallic complexes. A critical yet often overlooked factor is the non-innocent catalytic activity of the support. While this activity can enhance yields, it typically lacks stereocontrol, leading to racemic product formation. The issue is particularly relevant for silica and is commonly addressed by silanizing free OH groups to render them catalytically inert. Careful application of this surface modification is crucial, as revealed in a report by Corma et al. Herein, chiral Cu(II) complexes were anchored onto modified silica catalysts, namely SiO_2_ and mesoporous MCM-41 supports.^92^ The Cu-bisoxazoline complexes were prepared by reacting dimethyl undecenyl methylmalonate with (*R*)-2-amino-2-phenylethanol, followed by coordination with Cu triflate. The structure and chiral features of the complex were evaluated by liquid state NMR. The support was functionalized with mercaptopropyl groups to anchor the (*S*)-Cu-bisoxazoline complex using AIBN in toluene. Silanization with HMDS minimized free OH groups on the surface. The prepared catalysts were tested in the enantioselective Friedel–Crafts hydroxyalkylation of 1,3-dimethoxybenzene, achieving high conversions and enantioselectivities (up to 92% ee). The authors noted silanized silica performed better due to the nearly complete neutralization of surface hydroxyl groups, whereas MCM-41 retained about 30% of these groups after silanization, leading to lower ee values. Consequently, optimizing surface treatment to the target support, is an important parameter to consider when designing a chiral SAC (Figure 8).

**Figure 9:**
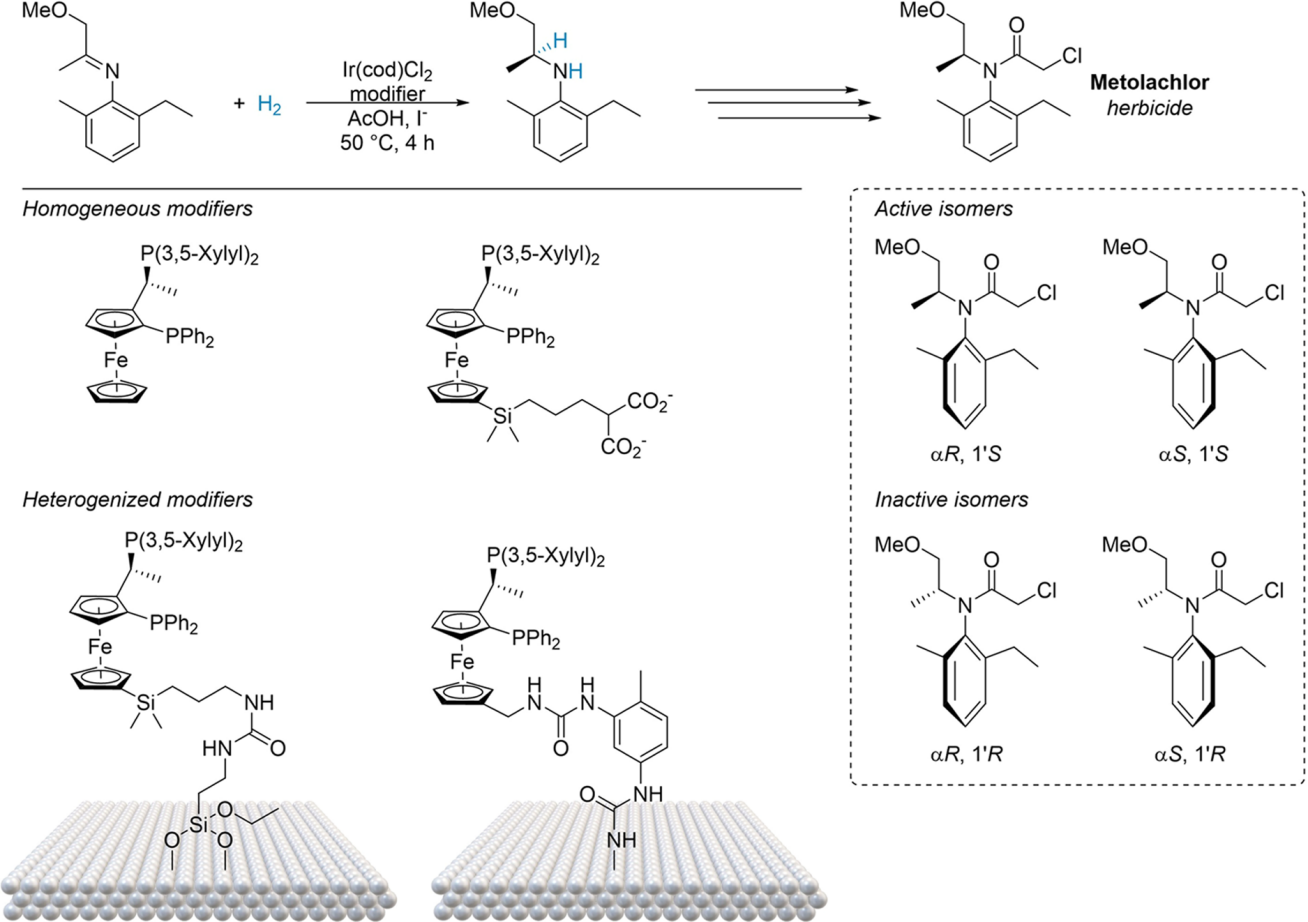
Metolachlor synthesis as a case study: key synthetic step (top), active and inactive isomers (bottom right), homogeneous and heterogenized chiral modifiers (bottom left). Adapted from refs (95−97).

The previous reports highlight the intricate balance necessary for effective chiral ligand immobilization in solid-phase asymmetric catalysis, which could impede the application of this approach to chiral SACs. This challenge is particularly relevant in industrial settings, where the production of (*S*)-metolachlor, a chiral tertiary amine herbicide, serves as a pertinent example. Its homogeneous and heterogeneous catalyst development is schematically depicted in Figure 9.^93^ Herein, intensive research identified a homogeneous Ir-Josiphos complex as the leading candidate; however, the catalyst was prone to base deactivation. In addition, the potential to improve catalyst recovery and limit purification steps served as the impetus to immobilize the catalytic species on solid supports. Various design strategies were proposed with one of the most successful employing OH or NH functionalized diphosphine modifiers anchored to various supports by diisocyanate linkers. Subsequent metalation was carried out with appropriate metal precursors, such as [Rh(NBD)_2_]BF_4_, [Rh(COD)]Cl_2_, or [Ir(COD)]Cl_2_ (where NBD = norbornadiene and COD = cyclooctadiene). Silica emerged as the most effective support, allowing the catalyst to rival the enantioselectivity of its nongrafted counterpart. Despite this, mass transport limitations caused the activity of the immobilized catalysts to fall short of the free catalysts, with turnover numbers (TONs) of around 12500 compared to over 600000 for the homogeneous system. Ultimately, it was deemed more efficient to remove the homogeneous catalyst by distillation - despite diphosphine ligand loss - than to continue developing the more expensive and less active heterogeneous variant, which offered no significant processing advantages.^94^ This case study reflects a recurring issue with tethered organometallic catalysts: improving enantioselectivity often comes at the expense of lower activity,^95−97^ a challenge chiral SACs may help overcome (see Section 4).

**Figure 10:**
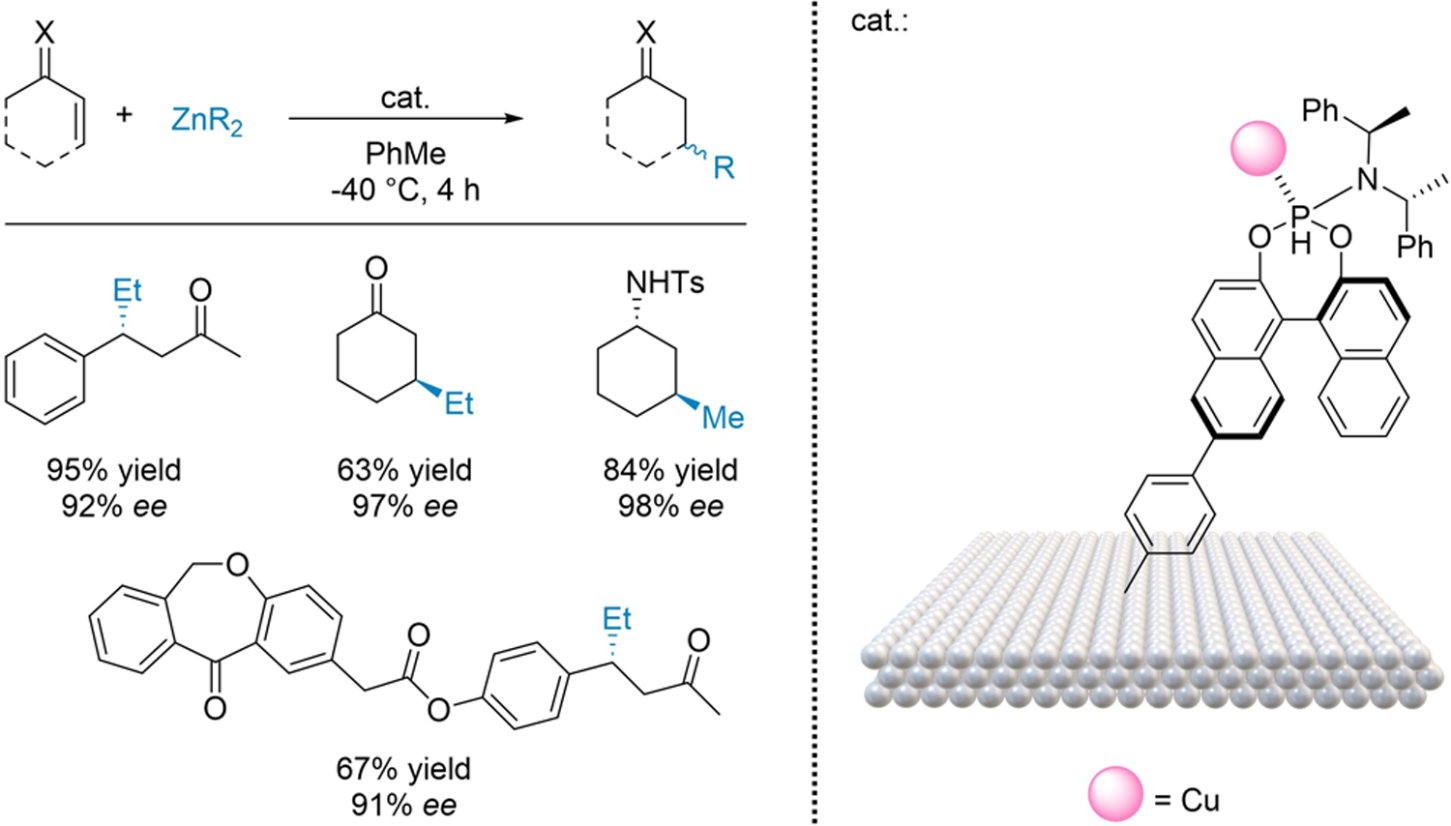
Chiral Cu(I)-phosphoramidite complex tethered on a (poly)styrene-divinylbenzene resin for the alkylzinc-mediated alkylation of imines and ketones. Adapted from ref (98).

Despite their shortcomings in batch settings, immobilizing the chiral inducing moiety onto the catalyst surface offers a promising approach to address insufficient modifier coverage in continuous flow systems. Wang et al. covalently grafted a Cu(I) phosphoramidite complex onto a polystyrene/divinylbenzene resin; to prepare the material, the authors first synthesized the phosphoramidite ligand by attaching a 4-vinylphenyl group at the 6′ position of the BINOL skeleton in the presence of Et_3_N.^98^ The ligand was metalated with copper(I) thiophene-2-carboxylate and grafted onto a polystyrene-divinylbenzene resin formed in situ from divinylbenzene, styrene, and AIBN in a toluene/water mixture. The hybrid metallaorganic material was thus applied to the asymmetric conjugate addition of organozinc reagent to imines and ketones, furnishing impressive yields of up to 95%, and ee up to 99% (Figure 10). Most notably, the catalyst maintained its excellent performance under flow conditions, delivering yields of up to 93% and 92% ee. Consequently, immobilization in this instance streamlined catalyst recovery and supported the scalability of the process to gram-scale under flow.

### 2.4 Confinement of Metal Catalytic Centers

The methods discussed thus far primarily focus on inducing chirality through chemical interactions between the substrate and the catalyst. In contrast, spatial confinement involves physically restricting the movement of reactants and intermediates close to the active catalytic site, typically utilizing the voids and channels of highly porous materials. This confinement alters the way molecules approach and interact with the catalytic site, creating a chiral microenvironment that favors specific orientations of reactants. The restricted space also stabilizes favorable transition states, reduces side reactions, and improves reaction efficiency by controlling diffusion, ultimately guiding the selective formation of one enantiomer over another. Porous materials for this approach can be commercially available, such as naturally occurring zeolites, which have well-defined structures and properties streamlining their application in chiral catalysis.^99^ However, their fixed pore size and limited tunability may impede the achievement of optimal enantioselectivity in specific reactions. In contrast, tailored synthesis of porous frameworks, such as COFs, MOFs, and certain silica supports (e.g., SBA-15, MCM-41) guarantees precise control over pore size.^100^ Indeed, the pore sizes of these materials are highly adaptable, allowing for customization to suit specific applications, particularly in MOFs and COFs.^101^ However, this process often involves trial and error, requiring the synthesis and testing of multiple derivatives with varying pore sizes to find the most effective one for a specific enantioselective reaction.

**Figure 11:**
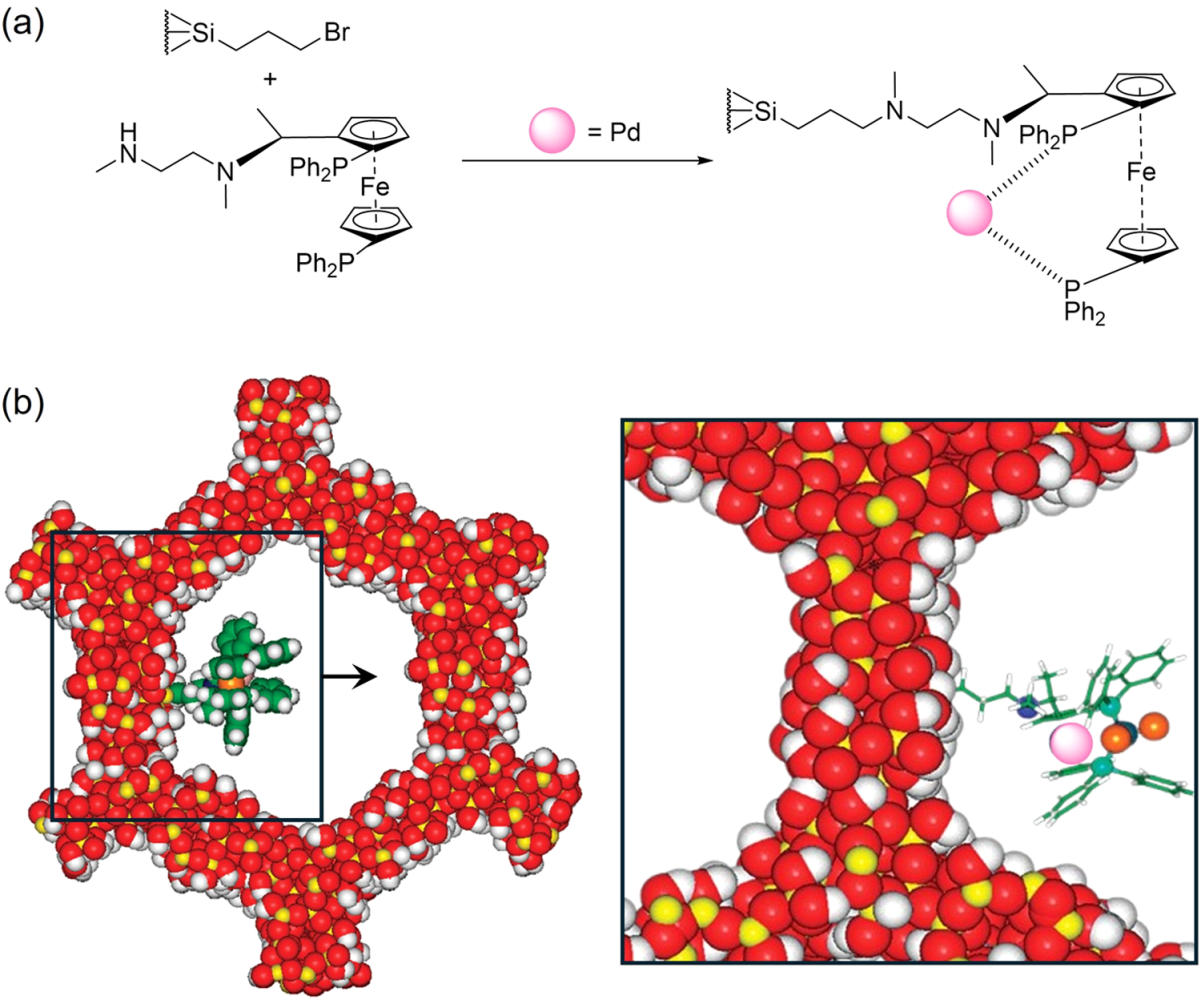
Confined Pd-DPPF complex in MCM-41 synthesis (a), and molecular view of the anchoring in the inner walls (b, left) and overall structure (b, right). Adapted from ref (102).

Regardless of the porous material utilized, the chiral catalyst must be incarcerated within the material. The methods already discussed in Sections 2.2 and 2.3 are excellent options to achieve this. In a pivotal publication, Thomas et al. confined chiral Pd and Rh diamino complexes within the inner walls or concave surfaces of MCM-41 using the tethering approach (Figure 11).^102^ In the first case, enantioselective control was achieved by controlling the space surrounding the metal center; in the second strategy, the concavity of the pore generated restricted access to the reactants, controlling the spatial configuration of the products. The materials were prepared by functionalizing MCM-41 with the bidentate ligand (1*R*,2*R*)-(+)-1,2-diphenylethylenediamine (DPPF) through 24-h reflux. Metal anchoring was subsequently achieved by impregnating the functionalized MCM-41 with Pd or Rh precursors and AgBF_4_ as a counterion. Single-crystal X-ray diffraction (XRD) confirmed the spatial arrangement of the tethered complex with significant steric hindrance noted around the square planar Rh catalytic center. The synthesized Pd and Rh catalysts demonstrated an ee of 92–96% in the asymmetric hydrogenation of α-cinnamic acid and methyl benzoyl formate.

**Figure 12:**
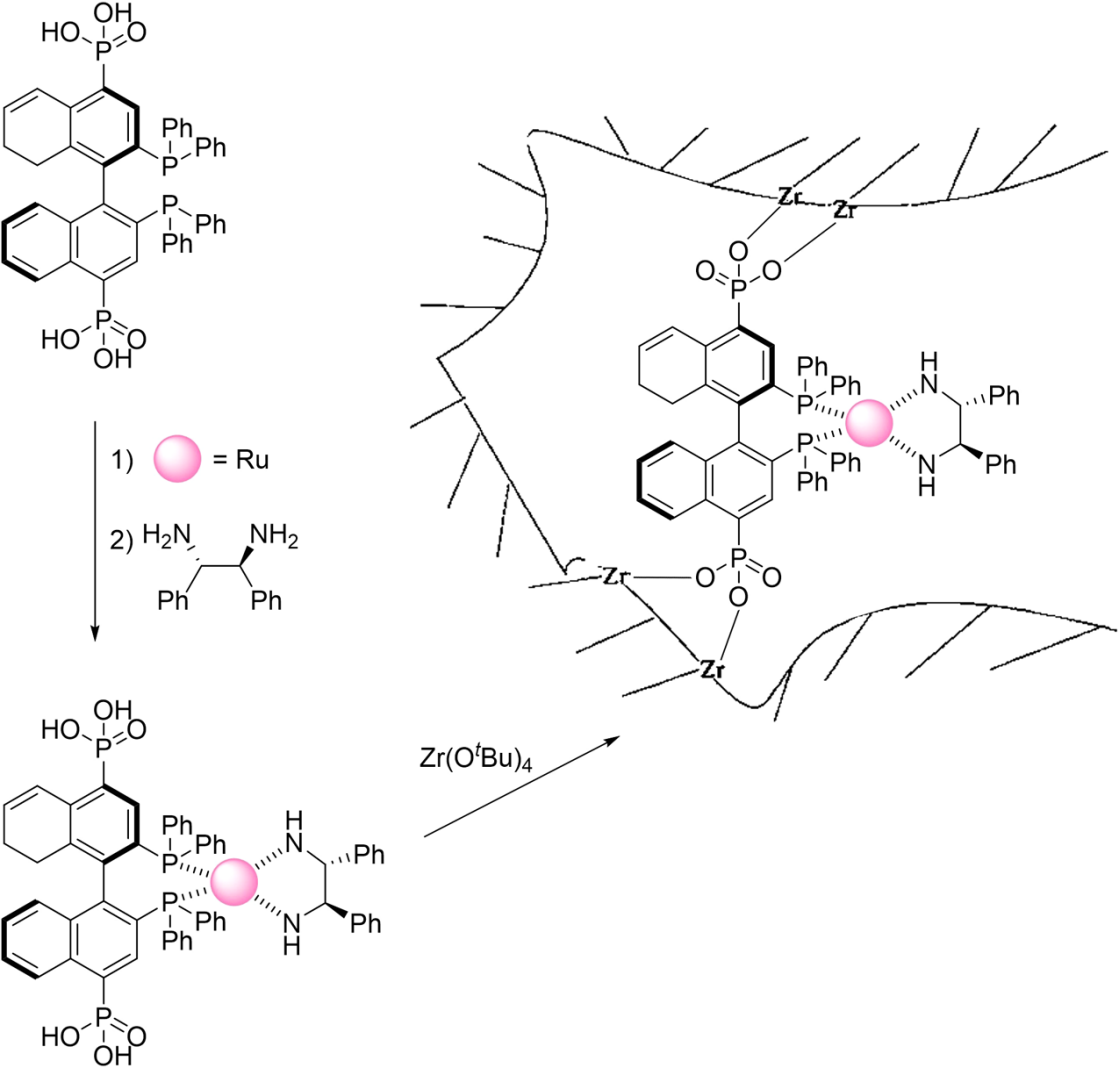
Ru-BINAP-DPEN confined in porous Zr phosphonate. Adapted from ref (103).

As alluded to previously, a crucial factor governing enantioselectivity and activity in confined catalysts is pore dimension. Hu et al. explored this by embedding a phosphonic acid-ruthenium complex within the cavities of porous zirconium-functionalized polymers. The synthesis, depicted in Figure 12, involved three steps: first, the Ru-2,2′-Bis(diphenylphosphino)-1,1′-binaphyl (Ru-BINAP) intermediate was prepared by reacting a Ru precursor with BINAP. Next, this intermediate was coordinated with (*R,R*)-diphenylethylenediamine (DPEN) to form the chiral Ru-BINAP-DPEN complex. Finally, the complex was integrated into a zirconium phosphonate framework through reflux with Zr(OtBu)_4_, yielding Zr[Ru(BINAP)(DPEN)Cl_2_]·4H_2_O. Two catalysts with wide pore distributions were generated with their chirality validated by liquid-state NMR analysis of the intermediates.

These hybrid organic/inorganic materials exhibited high activity and enantioselectivity in the selective hydrogenation of β-ketoesters, achieving up to 95% ee and quantitative yields, surpassing the performance of their homogeneous counterparts. This excellent activity is attributed to the bulkiness of the ruthenium complex, which selectively interacts with specific facets of the starting material, and the spatial control exerted by the confinement of active sites. The study further highlighted the importance of pore size: the version with a larger pore size showed inferior enantioselectivity compared to the smaller one.^103^ In a follow-up report, closely related catalysts were also tested in the hydrogenation of aromatic ketones, yielding similar results.^104^

**Figure 13:**
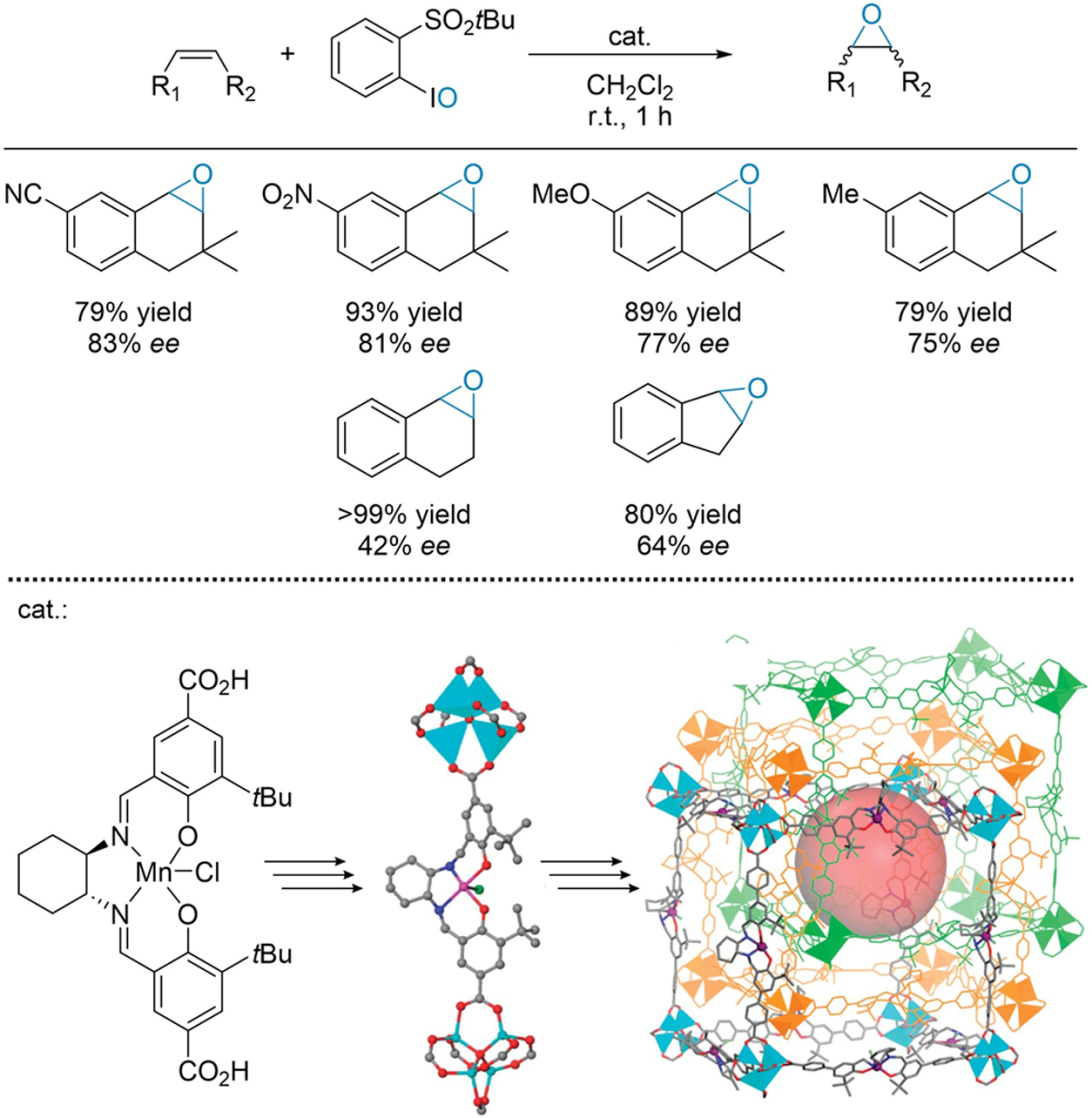
Mn-salen based MOF for the enantioselective epoxidation of alkenes. Adapted from ref (105).

Strategic channel size manipulation enhances enantioselectivity not just in hydrogenation, but also in various organic transformations, particularly when accommodating bulkier reactants. In this context, Song et al. developed a family of isoreticular Mn(II) salen-based chiral MOFs with an exact control of the cell dimension, and consequently open channel size (Figure 13).^105^ Enantiopure Mn-Salen dicarboxylic acid ligands were synthesized by Schiff base condensation of (*R,R*)-cyclohexanediamine and hydroxybenzaldehyde derivatives, followed by metalation with a manganese salt and *in situ* air oxidation to yield Mn(III) complexes. These were then reacted with Zn(NO_3_)_2_·6H_2_O at 80–90 °C for 96 h to yield single crystals of the desired MOFs. The chirality of the intermediates was confirmed by NMR, and the final 3D structure, featuring [Zn_4_(μ_4_-O)(carboxylate)_6_] units, was deduced by single-crystal XRD. Catalytic tests on the asymmetric epoxidation of various alkenes showcased the materials’ exceptional activity and enantioselectivity, achieving yields of up to 99% and ee of 42–92%. Notably, the enantioselectivities across all MOFs were broadly comparable and largely independent of pore size. However, reaction rates in small pore or interpenetrated structures were limited due to restricted diffusion of reactants and products. In contrast, materials with larger channel sizes exhibited comparable activity to the homogeneous benchmark catalyst, free from diffusion limitations.

**Figure 14:**
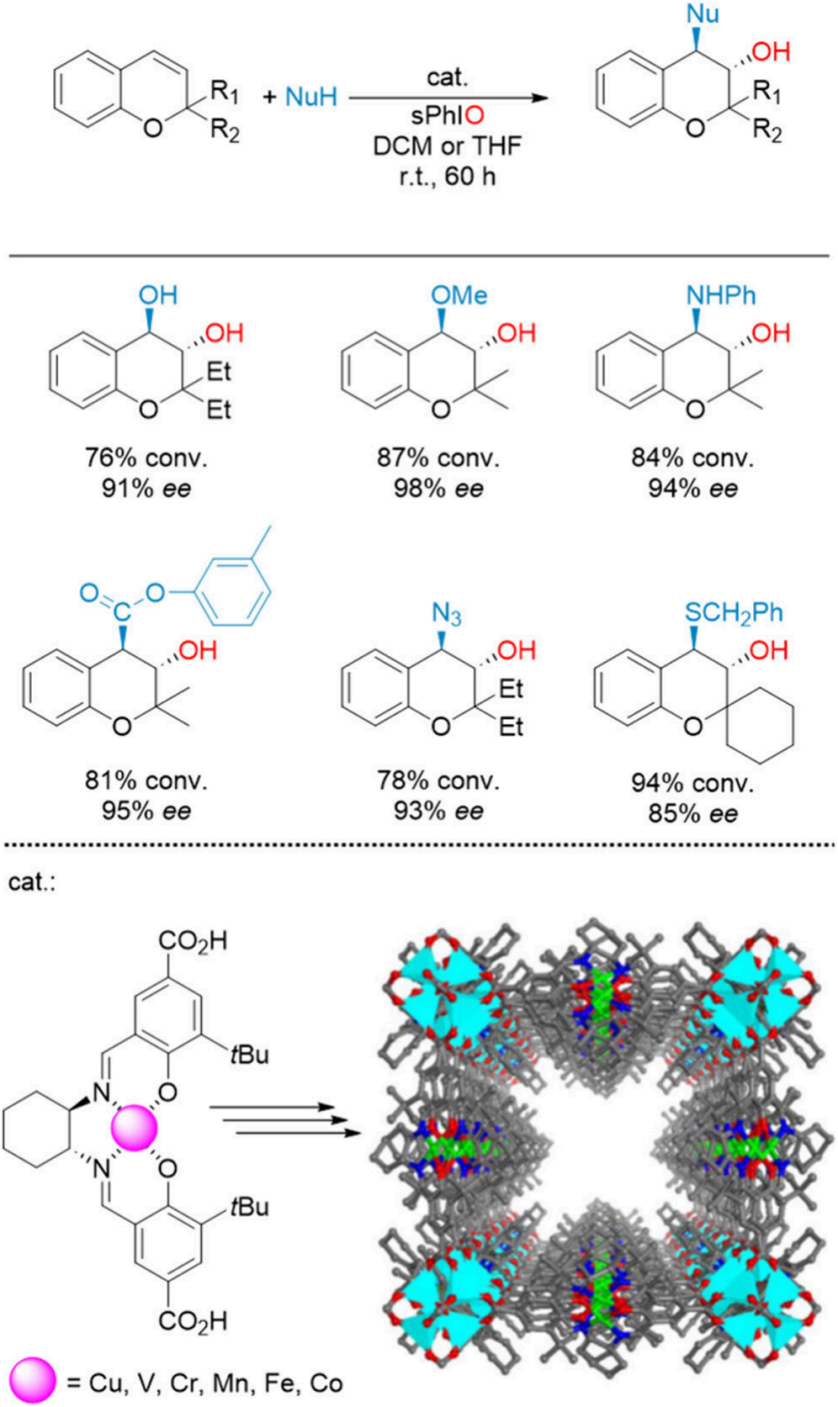
Metallosalen-based COF for the asymmetric cascade epoxidation-nucleophilic ring opening of chromenones. Adapted from ref (106).

One notable feature of MOFs and COFs is their ability to incorporate multiple distinct metal active sites. These multivariate platforms allow for the precise and reproducible spatial arrangement of different building units, to create multimetallic species, demonstrating enhanced enantioselectivity and efficiency compared to traditional monometallic materials. Xa et al. utilized this feature to construct mono-, bi-, and trimetallic metallosalen-based chiral MOFs using a combination of base metals such as Cu, V, Cr, Mn, Fe, and Co.^106^ First, the metallosalen-derived dicarboxylate ligands were synthesized by reacting the COOH-functionalized salen moiety with the corresponding metal salts at room temperature. Then, Zn insertion in the structural nodes was achieved by heating a mixture of Zn(NO_3_)_2_·6H_2_O and the ligand. Finally, bimetallic and trimetallic catalysts were obtained by mixing equimolar amounts of single crystals of the monometallic counterparts. Solid-state circular dichroism spectra of the materials, constructed from (*R*)- or (*S*)-enantiomers of the ligands, are mirror images of each other, confirming their optical purity. These catalysts demonstrated broad nucleophile tolerance (amines, azides, and water) in the tandem epoxidation-nucleophilic ring opening of alkenes. Exceptional enantioselectivities, up to 99% ee, were attributed to the 2-fold structure (Figure 14) of the material enabling cooperative interaction between adjacent metallosalen units.

**Figure 15:**
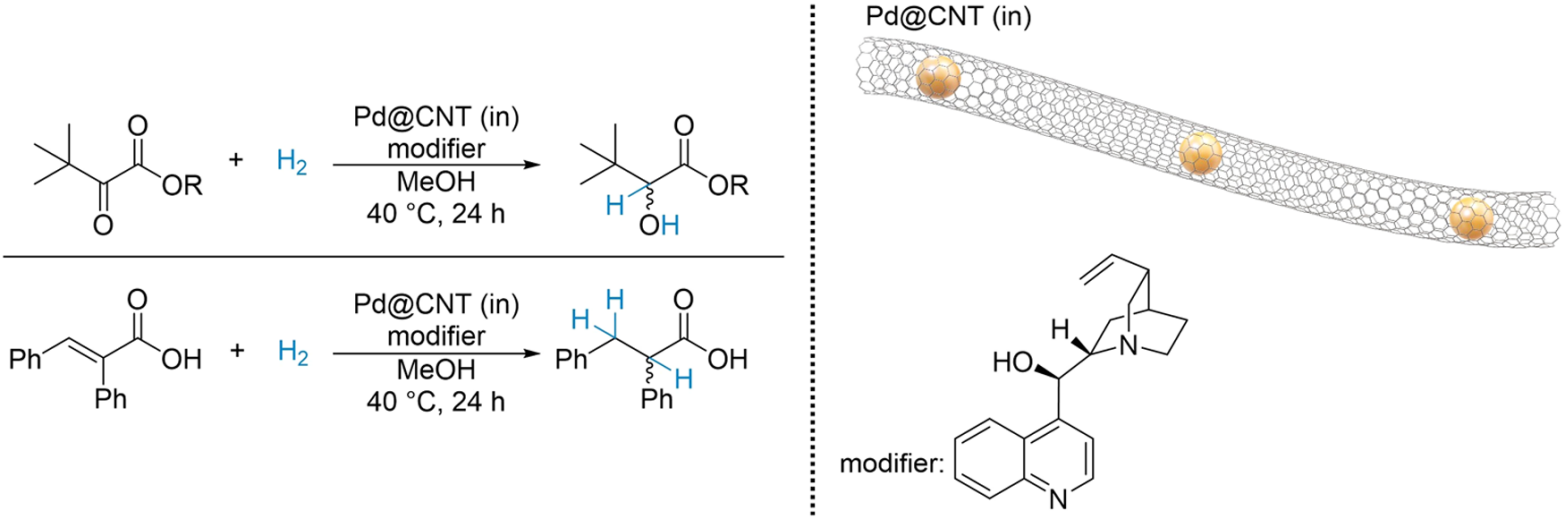
Cinchonidine-modified confined Pd NPs in carbon nanotubes for the hydrogenation of α-cinnamic acid and methyl benzoyl formate. Adapted from refs (107, 108).

The simplicity and effectiveness of the chiral modifier approach means attempts have prompted its integration with spatial confinement strategies. These methods rely on two key factors: (i) spatial control of the prochiral substrate conformation through encapsulation of metal active sites within the host support, and (ii) the molecular imposition of the desired substrate configuration by the chiral modifiers. For instance, Chen et al. synthesized and applied Pt nanoparticles to carbon nanotubes (Pt@CNTs) for the asymmetric hydrogenation of β-ketoesters, using cinchonidine as a chiral modifier. The catalyst was prepared by introducing the platinum precursor into open-ended CNTs at room temperature using ultrasonication, followed by stirring for 48 h. The suspension was then heated to 110 °C for 24 h to promote Pt insertion into the CNT channels. Afterward, the dried sample was reduced with a sodium formate solution, followed by filtration and drying to obtain the solid catalyst. Catalytic tests revealed that confining the metal sites within the CNT pores resulted in significantly higher TON and ee compared to nanoparticles anchored on the surface. The chiral modifier was essential for controlling both reactivity and enantioselectivity, as the absence of cinchonidine led to racemic mixtures. The high activity and enantioselectivity were attributed to the unique properties of the CNT nanochannels, which effectively enriched the local concentration of both the chiral modifier and reactants.^107^ Using a similar approach, the same group developed a cinchonidine-modified Pd@CNTs catalyst for the hydrogenation of α,β-unsaturated carboxylic acids (Figure 15).^108^ Notably, all the catalysts were recyclable with consistent activity and enantioselectivity over nine cycles, although the chiral modifier had to be replenished after each cycle, reflecting the semi-heterogeneous nature of chiral modifiers.

**Figure 16:**
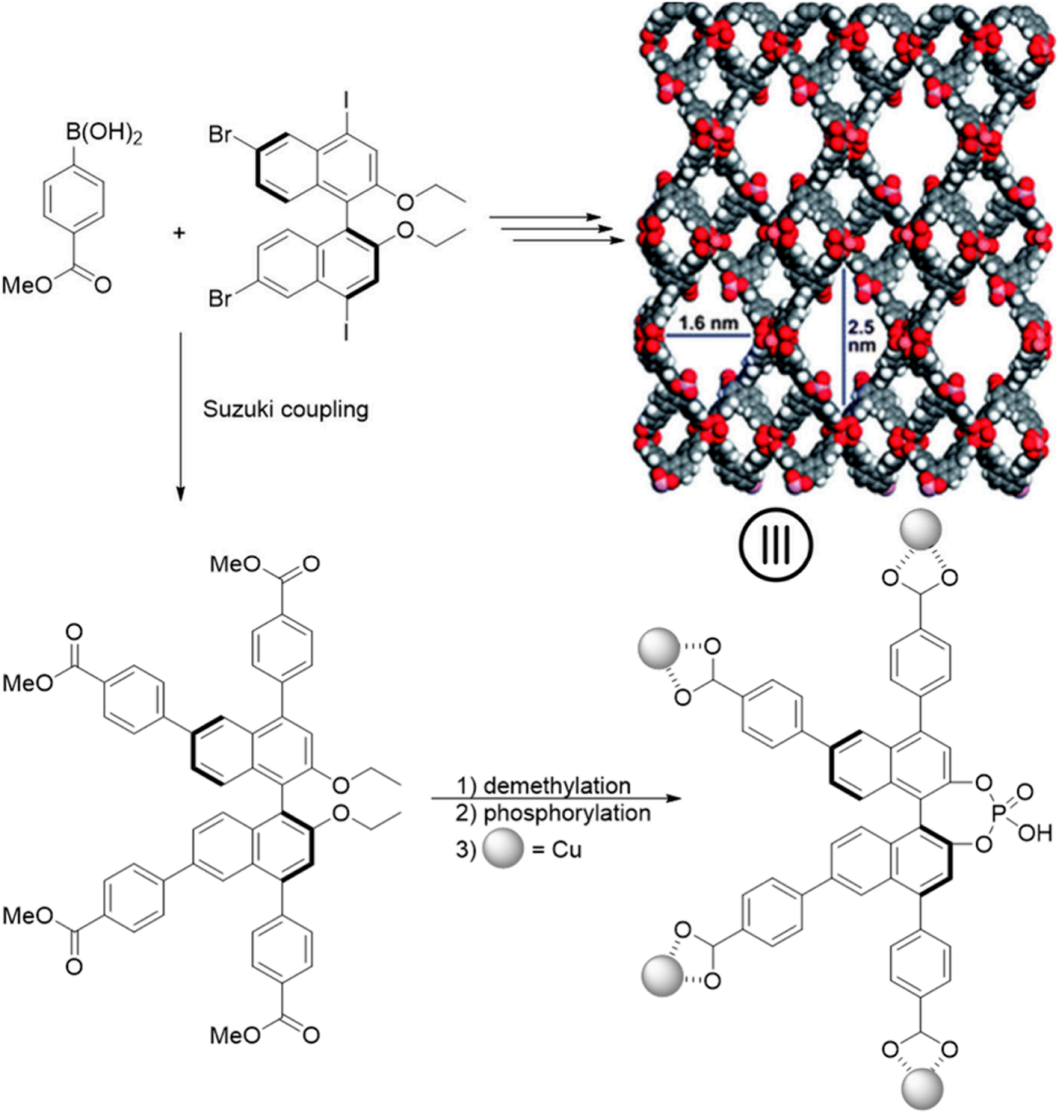
Cu-based MOF for the asymmetric Friedel–Crafts alkylation of indole with benzylic imines. Adapted from ref (109).

The works analyzed thus far showcased how spatial confinement in catalytic systems can enhance enantioselectivity in comparison to the equivalent homogeneous protocol. In certain cases, the effect of confinement can be strong enough to reverse the stereochemistry attained. This concept is exemplified in the work of Zheng et al., who employed [Cu_2_(carboxylate)_4_] and BINOL-derived ligands in MOFs, depicted in Figure 16.^109^ The enantiopure BINOL benzoic acid phosphate ligand was synthesized *via* Suzuki cross-coupling followed by acid catalyzed hydrolysis and phosphorylation. This building block was then reacted with Cu(NO_3_)_2_·2.5H_2_O at 80 °C for 2 days, yielding single crystals of the final catalyst. In this case, the carboxylate groups from four adjacent ligands coordinate to two Cu(II) centers, forming a [Cu_2_(carboxylate)_4_] secondary building unit. The chirality of the catalyst was retained throughout the chiral amplification process as established *via* liquid state NMRs. These MOFs were applied as Brønsted acid catalysts for the enantioselective Friedel–Crafts reaction between indole and imines, producing (*R*)-enantiomers with 6–44% ee, while the homogeneous catalyst yielded (*S*)-enantiomers with up to 89% ee. This reversal was attributed to the confined chiral environment within the MOFs cavity, which blocked certain reaction pathways and directed it through alternative transition states, mimicking enzyme-like stereocontrol. Though these findings illustrate spatial confinements potential to manipulate product chirality, the lower enantioselectivity attained, indicates potential for further cavity design optimization. Additionally, the detection of byproducts such as aryl(bisindolyl)methane suggests that intermediates may become trapped in the MOF framework, leading to undesired side reactions. This underscores the challenge of balancing selective catalytic pocket creation with efficient product release.

**Figure 17:**
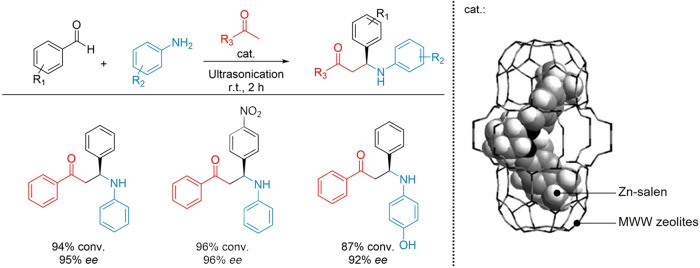
Encapsulated Zn-salen@MWW zeolites for the one-pot three-component synthesis of β-amino carbonyl under ultrasonic irradiation. Adapted from ref (110).

Finally, heterogeneous chiral protocols with metal centers confined in pores and cavities extend beyond simple binary reactions to encompass multicomponent processes. In a study by Lakhani et al., a chiral Zn(II)-salen complex was encapsulated on the surface of MWW-type zeolites in the one-pot three-component synthesis of β-amino carbonyls under ultrasonic irradiation.^110^ To prepare the catalytic materials, Zn-exchanged MWW zeolites were impregnated with an excess of chiral salen under reflux. Subsequently nonencapsulated salen moieties and Zn^2+^ free ions were removed by extensive washing. The chirality of the complex was determined by liquid state NMR analysis and its encapsulation inside the zeolite was confirmed by the reduction of surface area as detected via N_2_ physisorption. The encapsulation approach exploited the synergistic interaction between the zeolite acidic sites and the metallosalen molecular complex, resulting in high yields (94%) and stereoselectivities (up to 95% ee), alongside excellent recyclability over 5 reaction cycles (Figure 17).

## 3 Characterization of Chiral Heterogeneous Catalysts

In the previous sections, we highlighted innovative strategies for imparting stereoselective properties to solid catalysts, with a focus on approaches that could be translated to SACs. A crucial aspect of developing and optimizing any heterogeneous catalyst is obtaining detailed insights into their structure, composition, and activity. While a vast repertoire of characterization techniques is available to surface scientists for probing the physicochemical properties of catalysts, comparatively fewer can elucidate catalyst chirality.^111−115^ Most techniques currently available for characterizing chiral materials provide only indirect evidence of chirality, relying on the measurement of properties correlated with, rather than directly indicative of, the material’s chiral nature. Among these, only oriented circular dichroism and STM offer direct evidence of chirality. Furthermore, these techniques often require specialized instrumentation, highly trained operators, and carefully developed analytical protocols to ensure accurate and reliable data interpretation. These challenges are further exacerbated by the inherent complexity of SAC systems, where the unambiguous characterization of the single-atom nature remains an unresolved issue, lacking a universally accepted, bias-free methodology for precise identification and analysis. Table 2 provides an overview of the key methods, with brief descriptions and their application in different contexts. Further details can be found in the supporting references.

**Table 2:** Techniques for Determining the Chirality and Molecular Features of Heterogeneous Catalysts

Technique	Description	ref
Chiral surface plasmon resonance (CSPR)	Chirality identification via different excitation mechanisms of surface chiral plasmons.	(111)
(Oriented) circular dicroism (OCD)	Definition of chirality by different absorption of polarized light by the material. It can be oriented if the sample is placed in a certain orientation.	(112)
Single-crystal adsorption calorimetry (SCAC)^a^	Chirality detection via quantification of binding energies, which vary for (enantioselective) binding modes.	(115)
Scanning-tunneling microscopy (STM)	Atomic level recognition of the chirality via direct imaging.	(40)
Near-edge X-ray absorption fine structure (NEXAFS)^a^	Molecular orientation determination from the variation in the π* intensity associated with peaks as a function of the photon incidence angle, θ	(113)
Temperature-programmed desorption (TPD)^a^	Different desorption of chiral modifiers leads to peaks shift between enantiomers.	(40)
Reflection-adsorption infrared spectroscopy (RAIRS)^a^	Change of molecular features due to the chiral superstructures formed upon the adsorption of chiral modifiers.	(114)

a Particularly useful in the case of chiral modifiers.

The techniques described above can be complemented by the implementation of *in situ* or *operando* measurements to continuously monitor the enantioselective stability of the catalyst. However, developing these techniques is not straightforward, and to the best of our knowledge, no direct methods have been specifically designed for this purpose. Nonetheless, other spectroscopic techniques can provide indirect confirmation. Thus, in an operando ATR-IR spectroscopy study, the adsorption of cinchonidine on Pt@Al_2_O_3_ and Pt@C catalysts during the asymmetric hydrogenation of ketopanctonolactone was investigated. The results indicated that cinchonidine predominantly adsorbs on Pt nanoparticles showing negligible interaction with the support. Moreover, the IR signal intensity revealed that the quinoline moiety of cinchonidine tilts along its short axis during the reaction, which correlates with the observed enantioselectivity.^116^

Alongside advanced characterization techniques, computational approaches, particularly those based on Density Functional Theory (DFT), have been pivotal in advancing the understanding and design of SACs. Today, DFT enables the precise modeling of electronic properties of single metal atoms, their interactions with supports, and their dynamic interactions with reactants.^117,118^ For example, recent studies have demonstrated how DFT can elucidate critical aspects of SACs behavior, such as the stabilization mechanisms of single atoms on various supports and their unique catalytic performance compared to nanoparticle counterparts in a variety of important chemical transformations.^119^ Moreover, DFT-guided investigations into charge transfer processes and adsorption energies have unveiled key factors that govern the activity and selectivity of SACs in complex reactions.^120,121^

In this context, DFT can play a crucial role in the design of chiral SACs, but additional complexities arise from the properties of these catalysts. Beyond the factors outlined earlier, one of the primary challenges lies in modeling stable and reliable chiral surfaces that accurately reflect the real structure of the catalyst. In this context, theoretical models should be capable of distinguishing electronic (and geometric) effects between the chiral and nonchiral portions of the catalyst. Such precision introduces significant computational demands, which is nonetheless a commonly encountered issue for extended and complex surface simulations, where the trade-off between accuracy and computational cost becomes a critical issue. To address this, an innovative strategy recently developed by Pacchioni et al. involves approximating the extended surface with a small organic molecule that effectively captures the active site’s steric and electronic characteristics.^121^ This “molecular analogue” approach has demonstrated high fidelity in reproducing the behavior of the periodic system while substantially reducing computational costs.^122^ Future advancements could refine this methodology, enabling the simulation of increasingly complex chiral SAC systems using molecular chiral analogues with greater efficiency.

Finally, DFT can also be applied to investigate transition states, intermediates within catalytic cycles, and their interactions with the catalyst structure. However, this presents a challenge for DFT simulations, particularly in the case of chiral transition states, where the associated computational costs are significantly higher.^123,124^ To address these limitations, the integration of DFT with artificial intelligence and machine learning protocols offers a promising pathway. Such data-driven approaches, such as principal component analysis, K-means clustering, linear combination fitting, and neural networks, hold potential for efficiently handling large data sets, improving the accuracy of analyses, and substantially reducing computational demands.

## 4 Toward Single-Atom Catalysts for Asymmetric Synthesis

**Figure 18:**
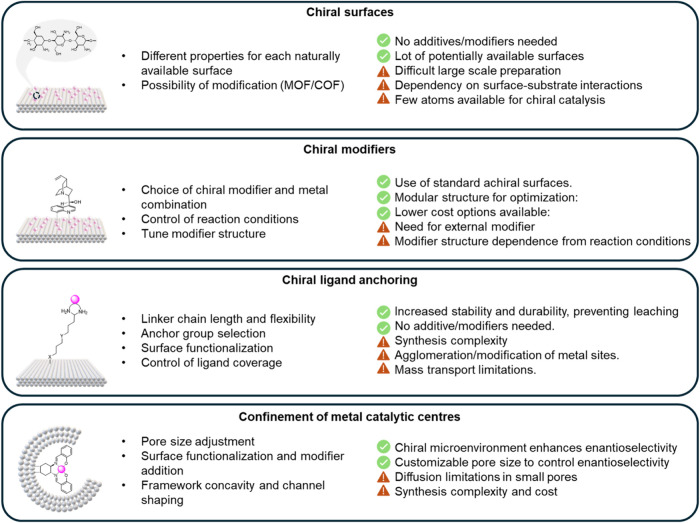
Potential chiral SAC catalyst design strategies with their tuning features (left), advantages, and disadvantages (right).

To date, the design of chiral SACs for enantioselective catalysis has not yet been fully realized, and we are far from achieving a true single heterogeneous surface capable of efficiently catalyzing asymmetric reactions. Nevertheless, the strategies described above for traditional (NPs-based) heterogeneous catalysts (Section 2) could be readily adapted to make chiral SACs, as illustrated in Figure 18.

### 4.1 Chiral Supports

The utilization of inherently chiral surfaces presents a highly effective strategy for designing enantioselective single-atom catalysts that, to the best of our knowledge, has yet to be explored. This approach avoids potential steric hindrance or competition issues, as the metal is hosted solely on the chiral surface. The benefits are intrinsic: the introduction of a chiral surface into the reaction environment controls the enantioselectivity of the process, while intrinsic features of SACs ensure maximized metal atom utilization and resistance to metal leaching, thereby boosting catalytic activity, recyclability and stability of the system in comparison to conventional nanoparticle catalysts.^125^ Most importantly, every single metal atom of a SACs will be in contact with the support meaning racemic product formation will be suppressed.

For these benefits to fully materialize, it is essential to consider and address certain potential pitfalls. One critical issue is that while the metal atom sites may be inherently stable in SACs, the chiral surface itself may lose or even invert its chirality during metal single-atom deposition or the catalytic reaction. In this context, molecular-level deposition techniques, such as atomic layer deposition (ALD), or other mild methodologies, can effectively minimize the risk of altering or disrupting the chirality of the support. Moreover, the proximity between the metal center and chiral moiety of the support is crucial for achieving concerted catalytic activation and enantioselectivity.^126^ If the distance between these sites is not optimized—whether too close or far apart—catalyst effectiveness is undermined and racemate production may occur. To mitigate these challenges, the resurgence of interest in synthetic 2D chiral supports should be taken advantage of. This could be key in preserving the desired chirality as certain examples have shown enhanced stability at elevated temperatures and harsh reaction conditions. Another transformative advantage of these synthetic materials over naturally chiral counterparts lies in their unparalleled tunability, enabling the precise engineering of chiral surfaces with tailored geometries and functional groups. This capability offers the potential to optimize the spatial relationship and fine-tune interactions between the metal center and the chiral moiety, paving the way for unprecedented control over catalytic processes.

### 4.2 Chiral Modifiers

This semi-heterogeneous approach is particularly appealing to chiral SACs, due to its minimal catalyst design requirements, pairing standard achiral SACs with a soluble chiral agent. However, its greatest strength is also its Achilles’ heel: the challenge of recycling and reusing the chiral catalyst-modifier system due to the reversible adsorption of the chiral modifier.^127^ To ensure complete coverage of all catalytic sites, the chiral modifier is typically used in significant excess (commonly 20 equiv).^128^ The stability of chiral modifiers under reaction conditions remains a concern, as they can degrade or desorb from the catalyst surface necessitating periodic replenishment. Additionally, pretreatment of the catalyst with the chiral modifier is often necessary to ensure reproducibility.^42^ These factors increase operational costs and contribute to waste, undermining the sustainability of the process. Moreover, the complex interaction between the chiral modifier and the catalyst surface complicates the design and prediction of effective modifiers as electronic effects, steric hindrance, and hydrogen bonding significantly affect enantioselectivity. Lastly, the limited substrate compatibility of certain chiral modifiers restricts their applicability and highlights the need for more universally applicable chiral modifiers.^118^

To address these issues, innovative strategies are required, with SACs offering a promising approach. Their well-defined and uniform active sites can potentially enhance the stability and effectiveness of chiral modifiers, ensuring consistent enantioselectivity. This precision could also allow for the use of smaller quantities of chiral modifiers to achieve complete coverage of active sites. However, this advantage may also introduce challenges such as elevated steric hindrance and site competition for the substrate, which are less likely in nanoparticles due to their higher exposed metal surface per cluster site. Therefore, it is crucial to carefully select appropriate combinations of chiral modifiers and SACs. One key consideration is the choice of support material; high surface area supports, with mesoporous structures or frameworks decorated with appropriate functional groups, can strengthen interactions between chiral modifiers and metal atoms, reducing leaching, and improving catalyst stability and performance. This can be complemented by designing “open” active sites, which remain readily accessible to both reactants and modifiers, thus addressing steric limitations while maintaining high catalytic activity.^129^ Such a tailored SAC design could redefine the compatibility and efficiency of modifiers in enantioselective applications. Equally important is reimagining the modifiers themselves. Historically, most chiral modifiers have been derived from naturally available compounds or minimally modified analogs, optimized primarily for interactions with metal nanoparticles. However, the structural and electronic properties of metal single atoms diverge significantly from those of nanoparticles, potentially suggesting a shift in modifier design. Future advancements should focus on engineering synthetic modifiers explicitly tailored to SAC systems, for example, by designing chiral ligands that promote dynamic adjustment of the local coordination sphere of the single atom. Moreover, the integration of computational methods, such as DFT, can accelerate the rational design of modifiers and SACs, providing predictive insights into optimal spatial configurations and chemical interactions between the nonchiral SAC and the modifier.

### 4.3 Anchoring of Chiral Ligands

Despite some examples existing in literature,^130,131^ the tethering of organometallic complexes and the exploitation of surface organometallic chemistry is a technically difficult strategy to achieve, due to the tendency of these system to undergo metal agglomeration, with a subsequent loss of the single-atom nature and decrease of catalytic activity, as already mentioned. These systems present intrinsic limitations due to their nature, namely length and nature of the linker, and its flexibility, that result to be independent from the dimensioning of the metal cluster size appropriately.^28^ Thus, also in this case the distance between the chiral ligand and the active catalyst center as well as the nature of the tethering point among others play a key role and must be finely tuned to maximize catalytic activity and enantioselectivity. DFT has potential in the design of next-generation SACs with chiral ligands, enabling the prediction of optimal tethering geometries and anchoring points for chiral ligands. Furthermore, its ability to model intricate interactions between ligands, linkers, and catalytic centers can guide toward the minimization of the steric hindrance and unlock superior enantioselectivity, offering a predictive blueprint for the rational design of innovative SAC systems. In this context, COFs and MOFs emerge as a versatile platform, not merely as hosts but as customizable architectures that can enhance ligand positioning and catalytic performance through finely tuned pore environments. Their design can open up entirely new dimensions in the development of chiral SACs.

### 4.4 Confinement of Metal Sites

This approach effectively addresses challenges encountered in earlier strategies. By entrapping chiral moieties within pores, it prevents the leaching and degradation often seen with chiral modifiers. Since tethering groups are unnecessary to hold the chiral moieties in place, their chiral properties are preserved in this case. Overall, the design and stability of the catalytic system is simplified from the perspective of the chiral moieties. It is thus not surprising early implementation of this approach has already been reported.^132−134^ To further advance the development of SACs with confined chiral moieties, innovative design approaches can focus on optimizing pore design, refining synthetic techniques, and leveraging synergistic catalytic interactions. Also in this case, hierarchically porous materials, which integrate micro-, meso-, and macropores, offer a promising avenue for simultaneously enhancing chiral recognition and improving substrate diffusion. In addition, functionalization of pore walls with secondary interaction-enabling groups, such as those that promote hydrogen bonding or π-π stacking, provides a compelling opportunity to further enhance enantioselectivity and substrate anchoring. Overall, while the application of chiral SACs in heterogeneous catalysis remains in its infancy, the potential for these materials is vast, offering a unique combination of high catalytic activity and enantioselectivity.

## 5 Conclusion

The development of chiral catalysts for heterogeneous reactions is both industrially important and synthetically challenging. Research into the innate chirality of surfaces has highlighted the difficulties of using naturally chiral materials, which, despite their potential, often fall short in terms of catalytic activity. The introduction of chiral MOFs and COFs has offered a compelling solution by employing the principle of “chiral amplification”. This methodology has allowed chirality to propagate throughout the framework, thereby enhancing enantioselectivity while also providing spatial confinement that stabilizes transition states and restricts reactant movement. Furthermore, the strategic incorporation of chiral modifiers, such as tartaric acid and cinchona alkaloids, can create localized chiral environments on achiral surfaces, which have been demonstrated to significantly enhance enantioselectivity in hydrogenation reactions.

To capitalize on the potential of chiral SACs, further research is necessary to refine these strategies and establish more cohesive frameworks for their rational design. Future studies should focus on exploring novel chiral supports and modifiers that enhance stability and enantioselectivity while minimizing leaching and degradation. Furthermore, the development of robust methods for the synthesis and characterization of chiral SACs is essential to fully understand their catalytic mechanisms and optimize their performance in asymmetric synthesis. The chiral SAC catalysts developed to date primarily utilize Ru and Rh as metal centers, which are scarce, high costly, and toxic. Consequently, exploring alternative metals, with particular attention to those that are safer, more cost-effective, and earth-abundant, is essential for the synthesis of these catalysts. Finally, the integration of computational approaches could aid in predicting the behavior of chiral SACs and facilitate the identification of optimal designs tailored for specific catalytic applications,^135,136^ and collaboration between experimental and theoretical researchers could accelerate the discovery and implementation of new strategies for chiral SAC development. The journey toward the widespread use of chiral SACs in enantioselective catalysis is just beginning, yet it holds great promise for advancing synthetic methodologies in organic chemistry.
